# Environmental Micro(nano)plastic Exposure and Associated Human Health Risks: A Comprehensive Review

**DOI:** 10.3390/toxics14050442

**Published:** 2026-05-18

**Authors:** Weike Hu, Dongling Liu, Jianing Wang, Xia Huo, Xiang Zeng

**Affiliations:** 1 School of Public Health, Zhejiang Chinese Medical University, 548 Binwen Road, Hangzhou 310053, China; 2 School of Basic Medical Science, Zhejiang Chinese Medical University, 548 Binwen Road, Hangzhou 310053, China; 3 Laboratory of Environmental Medicine and Developmental Toxicology, College of Environment and Climate, Jinan University, Guangzhou 511443, China; 4 Zhejiang International Science and Technology Cooperation Base of Air Pollution and Health, School of Public Health, Zhejiang Chinese Medical University (ZCMU), 548 Binwen Road, Hangzhou 310053, China

**Keywords:** micro(nano)plastics, toxicology, bioaccumulation, exposure pathway, ecological risk, human health

## Abstract

Micro(nano)plastics (MNPs) represent a pervasive and escalating threat to global ecosystems and human health. This review provides a critical synthesis of MNPs’ exposure risks across marine, atmospheric, and terrestrial compartments, with a distinct emphasis on identifying cross-media linkages and methodological inconsistencies that limit current risk assessments. Within marine environments, pollution hazard indices reveal significant spatial heterogeneity, yet their utility is constrained by the absence of toxicity weighting and particle characteristic integration. Atmospheric exposure profiles show variable risks, and the MNPs’ concentration in indoor air (up to 15.8 particles/m^3^) is significantly higher than in outdoor environments, posing a greater inhalation risk to infants and children who spend more time indoors. A marked increase in MNPs’ concentrations within agricultural soils is identified, where the MNP content in mulched soils (average: 570.2 particles/kg) is more than twice that of non-mulched soils (259.6 particles/kg). Critically, studies have now detected MNPs within human tissues, including the blood, intestines, liver, kidneys, tonsils, and brain, highlighting an urgent need to elucidate their multi-organ toxicity mechanisms, with a novel synthesis of gut–brain axis disruption and transgenerational effects. By integrating exposure dynamics with mechanistic toxicity data, this review advances a cross-system framework that identifies priority research directions, namely standardized detection methodologies, combined pollutant toxicity, and cross-system toxicity mechanisms, which are essential for informing mitigation strategies amid this escalating public health crisis.

## 1. Introduction

Plastics, defined as synthetic or heavily modified natural polymers, represent a heterogeneous class of materials with diverse chemical compositions and environmental behaviors [1,2,3]. Since the 1950s, global plastic production has surged, reaching approximately 367 million tonnes in 2020, with projections exceeding 33 billion tonnes by 2050 [4]. Despite growing awareness, global plastic recycling rates remain below 10%, leading to massive accumulation in landfills and ecosystems, posing escalating threats to environmental and human health [5,6]. Larger plastic debris undergoes degradation via physical, photochemical, and biological processes, fragmenting into microplastics (MPs; <5 mm) and nanoplastics (NPs; <1 μm or <100 nm), collectively termed micro(nano)plastics (MNPs) [7]. Beyond the polymer matrix (e.g., PE, PP, PVC, PS, PET), MNPs contain diverse additives (e.g., plasticizers, flame retardants) and exhibit high specific surface areas, enabling them to adsorb co-occurring pollutants like heavy metals and persistent organic pollutants, acting as potent contaminant vectors [8,9,10].

MNPs have been ubiquitously detected across all environmental compartments, including oceans, rivers, lakes, soils, the atmosphere, and even remote polar regions [11]. Their environmental fate is governed by their physicochemical properties: denser particles tend to settle in sediments, while lighter ones float on water surfaces or become suspended in the air, facilitating long-range transport via wind, rainfall, and ocean currents [12]. Human exposure occurs predominantly through ingestion (of contaminated food and water) and inhalation (of airborne particles), with dermal contact as a secondary route [13,14]. Accumulating evidence has confirmed the presence of MNPs in various human tissues and biofluids, including the blood, lungs, intestines, placenta, testis, and even the brain, demonstrating their capacity to cross biological barriers and distribute systemically [15,16,17,18,19,20,21]. Concurrent with the growing recognition of widespread MNP contamination, an expanding body of toxicological research has revealed adverse effects across multiple organ systems. Animal models and in vitro studies have shown that MNPs can induce oxidative stress, inflammatory responses, metabolic dysregulation, and cellular apoptosis, with emerging evidence linking MNP exposure to cardiovascular diseases, intestinal barrier dysfunction, hepatic fibrosis, neurotoxicity via the gut–brain axis, and endocrine disruption affecting reproductive and thyroid function [22,23,24,25]. Moreover, the ability of MNPs to act as vectors for adsorbed environmental pollutants can amplify their combined toxicity, further complicating risk assessments [10,26,27].

Despite these advances, significant knowledge gaps remain. MNPs were first identified in marine settings two decades ago, but the current scope of their impact today is truly alarming [3]. Environmental risk assessments (using the Pollution Load Index (PLI) and Polymer Hazard Index (PHI)) and most toxicological studies do not reflect real-world chronic, low-dose exposure to complex, aged MNP mixtures. Second, comprehensive assessments of atmospheric MNP exposure across different age groups and regions are lacking, and the pathways and impacts of MNPs in agricultural systems (via plastic film use, wastewater irrigation) require clearer elucidation to understand food chain contamination [11,28,29]. Therefore, this review aims to synthesize the latest data on MNPs’ occurrence and exposure risks in marine, atmospheric, and terrestrial environments, critically evaluating current methodologies; integrate mechanistic insights into MNPs’ toxicity across organ systems, highlighting emerging concerns like the gut–brain axis and transgenerational effects; and propose a cohesive framework linking environmental exposure to health outcomes, identifying priority research directions and informing evidence-based mitigation strategies.

## 2. Exposure Status of Microplastics in the Environment

### 2.1. Sources of MNPs

**Figure 1:**
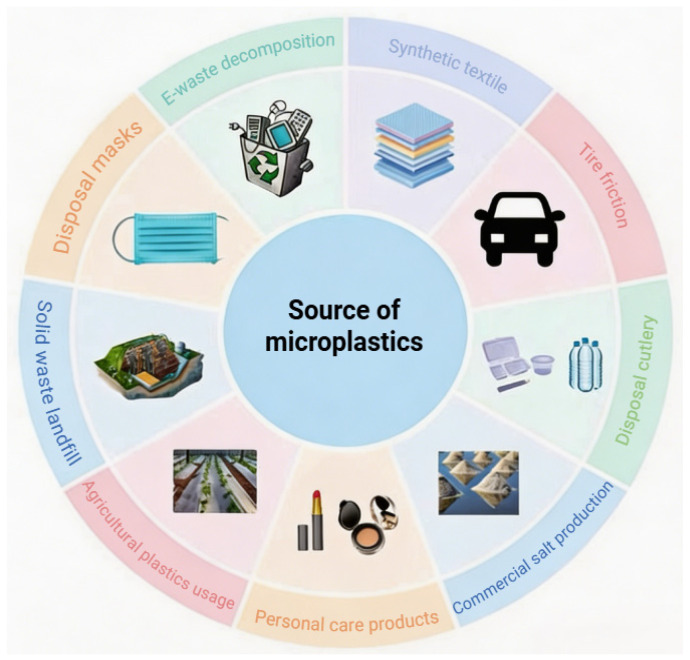
Major sources of environmental micro(nano)plastics (MNPs). MNPs are released into the environment from various sources, such as electronic waste decomposition, synthetic textile wear during washing and use, tire wear from road traffic, improper solid waste landfilling, fragmentation of agricultural plastic films, and sea salt production processes. These sources contribute to widespread contamination across terrestrial, aquatic, and atmospheric compartments.

Different styles and types of plastic products have emerged, updated, and grown rapidly in recent years (Figure 1). Notably, China has been the largest plastic producer globally, accounting for approximately one-third of total production [30]. The sources and distribution of microplastics are so extensive that it is need to carry out targeted efforts to prevent and control microplastic immediately (Figure S1).

As illustrated in Figure 1, existing studies indicate that MNPs originate from diverse sources and are widely distributed across various environments [31]. Key contributors include landfill solid waste, synthetic textiles, disposable masks, bottled water and plastic cups, sea salt production, agricultural plastic film, tire–road wear, and cosmetics and personal care products. Landfills, which receive an estimated 21–42% of global plastic waste, represent a major pathway for MNPs’ release [32]. Moreover, synthetic textiles release substantial amounts of microfibers throughout their lifecycle, entering ecosystems via wastewater [33]. In addition, disposed masks break down into MNPs through weathering and leaching. Moreover, the widespread use of single-use plastic food containers further exacerbates this issue. Furthermore, sea salt production also introduces considerable microplastic contamination. Notably, agricultural plastic films significantly contribute to soil MNPs’ accumulation, with national averages reaching 83.6 kg/hm^2^ and as high as 230.9 kg/hm^2^ in Xinjiang province, China; MNP levels in mulch-covered soils were more than double those in uncovered soils (570 vs. 260 particles/kg). Interestingly, tire wear particles (TWPs) are dislodged during use and enter dust, soil, and the atmosphere. Finally, microbeads in personal care products are a notable source, with one wash potentially releasing approximately 94,500 microfibers. These diverse sources release MNPs into various environmental matrices. Given that water bodies act as the ultimate sinks for most terrestrial runoff and plastic leakage, the following section evaluates the exposure status in marine environments.

### 2.2. Exposure to Microplastics in the Marine Environment

Unrecycled and unprocessed plastic waste accumulates in estuaries, bays, straits, and coastal zones, and eventually enters the open ocean [34]. Microplastic contamination has become ubiquitous in marine ecosystems, reaching even remote polar regions and equatorial zones due to global ocean currents and wind-driven dispersal [35,36]. MNPs in the ocean can interpenetrate with surface freshwater and terrestrial environments through resuspension, precipitation, and surface runoff, leading to widespread human and ecological exposure [37,38]. Atmospheric resuspension of MNPs poses a significant threat to human health, influenced by particle size, wind, humidity, precipitation, altitude, and human activities. Smaller particles exhibit greater susceptibility to resuspension due to their lower settling velocities. Wind speeds of >5 m/s are sufficient to resuspend MNPs from dry ground surfaces. Pedestrian traffic can cause an immediate three- to fivefold increase in ground-level concentrations. Vehicle traffic contributes to tire and road wear particle (TRWP) resuspension, accounting for up to 40% of traffic-related emissions. Notably, low humidity (<40%) reduces particle adhesion, resulting in resuspension rates in arid regions being two or three orders of magnitude higher than in humid areas. Rainfall temporarily deposits 50–70% of airborne MNPs, but these particles can become resuspended once the conditions are dry.

#### 2.2.1. The Typical Assessment Methods of Microplastic Pollution

To systematically evaluate microplastic pollution, the Pollutant Load Index (PLI, which quantifies microplastics’ abundance and distribution) method and the Polymer Hazard Index (PHI, which assesses the toxicity risk according to the plastic polymer types) method are used to assess the occurrence of microplastics in the ocean and the risk level of microplastic marine environmental pollution [39,40].

Table 1 summarizes data from diverse global regions, showing significant spatial variability. However, these indices have key limitations: they lack toxicity weighting, ignore critical particle characteristics (size, shape, aging), and depend on inconsistent baseline data and sampling methods, hindering cross-study comparison.

#### 2.2.2. Transmission of MNPs in the Marine Food Chain

**Figure 2:**
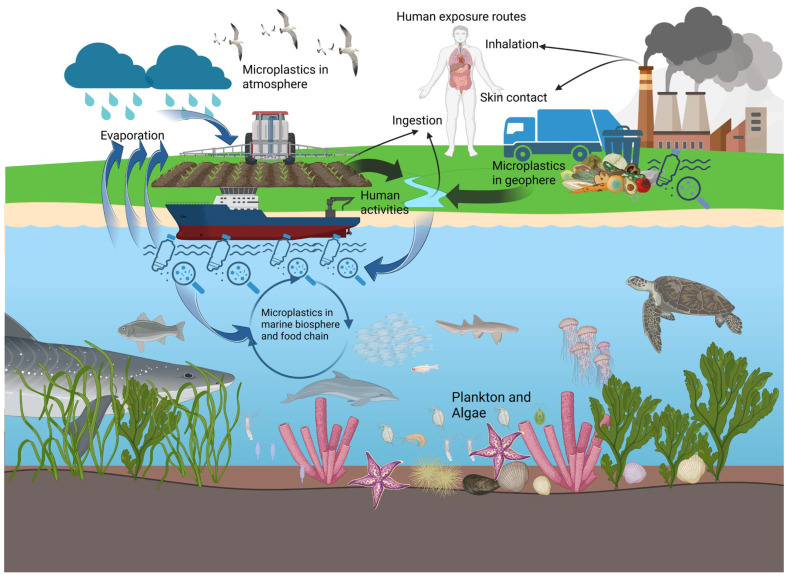
Potential sources of microplastics through the food chain.

MNPs, widely distributed in aquatic ecosystems, are ingested by diverse marine organisms (algae, crustaceans, fish, plankton, marine mammals, arthropods, and seabirds) and can accumulate in humans via the marine food chain (Figure 2) [54,55]. Interestingly, MNPs in human feces are similar to those present in the gastrointestinal tracts of marine organisms according to the Fourier transform infrared spectroscopy (FTIR) method [56].

**Figure 3:**
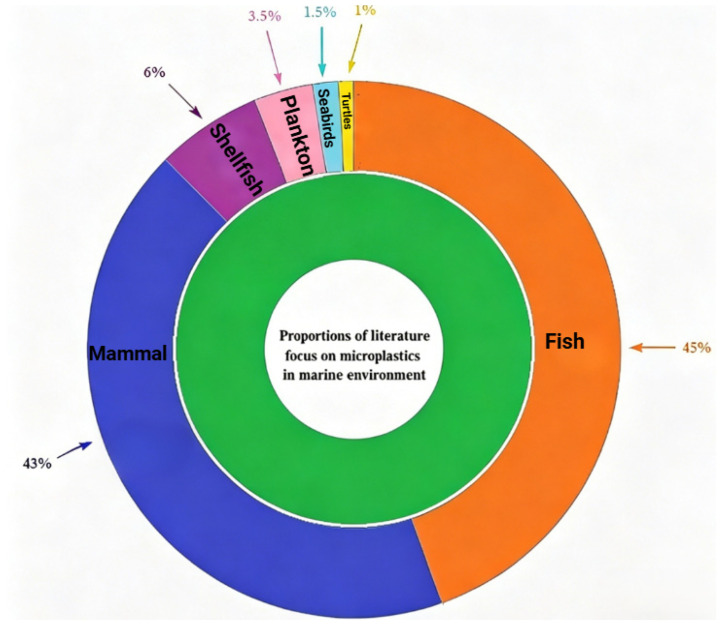
Sunrise chart of the proportions of literature focusing on microplastics related to the marine food chain.

MNPs in the marine food chain are predominantly studied in human-consumed species such as fish, marine mammals and shellfish. Plankton (mainly copepods) constitute the foundation of the marine food chain and may act as the entry point for MNPs into the marine food chain [57]. Notably, they exhibit marked disparities in their capacity to ingest and excrete MNPs [58]. Higher trophic consumers, such as seabirds [59] and fish [60], transfer MNPs, with the concentration potentially increasing along the food chain [61]. However, these effects are not universally observed and vary by trophic levels [62,63]. Controversy remains regarding significant bio-amplification, influenced by plankton diversity, microplastics’ properties (size, polymer), and organismal uptake/excretion capabilities. In this review, we collected statistical data from the literature published on the Web of Science between 2022 and 2025 concerning aquatic organisms, microplastic pollution, and health risks, as shown in Figure 3.

#### 2.2.3. Combined Effects of MNPs and Other Environmental Pollutants

MNPs threaten human health via the food chain and, due to their small size and high specific surface area, they significantly amplify the overall risk when co-occurring with pollutants such as persistent organic pollutants [64], heavy metals [65], pathogenic bacteria, or viruses [66]. The underlying mechanism involves several key processes. In aquatic environments, MNPs preferentially adsorb organic molecules, metal ions, and proteins, forming an environmental corona or biofilm after microbial colonization. Existing research shows that environmental microplastics undergo aging driven by physical factors like erosion and ultraviolet radiation. This aging generates oxygen-containing functional groups (e.g., hydroxyl [-OH] and carboxyl [-COOH]) on their surfaces, enabling complexation with heavy metals, while surface wrinkles and fractures increase the specific surface area and enhance adsorption via the van der Waals force [67]. Aging also increases negative surface charge, strengthening electrostatic attraction [68].

Following environmental corona formation, microbial colonization can further transform this layer into a biofilm. Microorganisms attach to aged MNPs’ surfaces and secrete extracellular polymeric substances (EPS), forming a mucous matrix [69]. This biofilm enhances the microplastics’ capacity to concentrate pollutants and facilitates the mobility of adsorbed heavy metal, while biofilm microorganisms can transform heavy metal speciation, potentially increasing biological toxicity.

The adsorption capacity of MNPs is influenced by physicochemical properties, including surface area, internal amorphous structure, hydrophilicity/hydrophobicity, shape, and particle size [70]. Key environmental factors, such as water pH, ionic strength, and dissolved organic matter concentration, further modulate these interactions [71]. Consequently, a detailed understanding of these polymer-specific physicochemical properties is therefore essential for accurately assessing the health risks of MNPs, both as isolated contaminants and as vectors for co-contaminants.

While the ocean is a major reservoir, MNPs are not confined to the hydrosphere. Their low density and aerodynamic properties facilitate long-range atmospheric transport, shifting the exposure risk from ingestion of seafood to continuous inhalation, as discussed in Section 2.3.

### 2.3. Current Status of Microplastic Exposure in the Atmosphere

#### 2.3.1. Methods for Atmospheric Monitoring and Exposure Assessment

The environmental fate of MNPs is not confined to aquatic systems; their low density and small size facilitate resuspension, leading to significant atmospheric transport and inhalation risks, as discussed in the following section. In the past decade, MNPs have been primarily regarded as marine pollutants. However, emerging studies increasing reveal significant microplastic contamination in the atmospheric environment. Synthetic fibers constitute over 90% of the atmospheric microplastics. Human exposure primarily occurs through inhalation and dermal contact. A comprehensive understanding of exposure characteristics across diverse regions and scenarios is essential for assessing and mitigating the associated health risks. The status of indoor and outdoor atmospheric microplastic pollution across various regions has been summarized in Table 2, noting that sampling methods varied by location, complicating direct comparisons.

To date, research on atmospheric microplastic (MP) concentrations predominantly combines passive and active sampling methods [48]. Passive sampling reports deposition rates (MP/m^2^/d), while active sampling measures particle counts (particles/m^3^). However, the absence of standardized methods for sampling, detection, and identification has resulted in limited comparability of the existing data. Establishing standardized sampling and analytical methods is therefore urgently needed.

#### 2.3.2. Indoor and Outdoor Exposure Characteristics and Risks

The current formula for estimating an inhalation exposure dose (MPs/kg-BW/d) via passive sampling is given by Equations (1) and (2) as follows [85].

EF_chronic_ = (F × ED)/AT (1)

where EF is the exposure factor, F represents exposure frequency (d/week × week/yr), ED is the exposure duration (yr), and AT is the average time.

D_INH_ = (C × IR × EF)/BW (2)

where D_INH_ is the daily inhalation exposure dose (MPs/kg-BW/d), C is the atmospheric microplastic concentration (items/m^3^), IR is the inhalation rate (m^3^/d), and BW is body weight (kg). Specific values for IR and BW are sourced from the Agency for Toxic Substances and Disease Registry (ATSDR) [86,87].

Using the median concentrations from global active sampling data and regional ranges from the Agency for Toxic Substances and Disease Registry (ATSDR), the default exposure factor (EF) value was set to 1 for indoor scenarios and 0.221 for outdoor scenarios [85].

Based on this framework, we quantified outdoor MNP exposure risks across several Chinese cities (Table S2) [88,89,90,91,92,93,94,95,96,97,98,99]. Comparative analyses of outdoor exposure and indoor exposure are provided in Figures S2 and S3. A key innovation of this review is the age-stratified inhalation risk assessment, revealing that younger age groups exhibit higher susceptibility to atmospheric microplastic exposure globally. Notably, atmospheric MP concentrations in China significantly exceed global averages, resulting in higher population exposure risks. Regional comparisons within China further reveal elevated concentrations in urban versus rural areas and coastal versus inland zones (Figure S4). These patterns likely reflect disparities in demographics, climate, industrial profiles, and environmental governance. Critical knowledge gaps persist in individual exposure assessments, particularly across diverse populations, age groups, and exposure scenarios. Collectively, these findings underscore the need for standardized MP measurement methodologies to enable robust cross-regional risk comparisons.

Atmospheric deposition eventually returns airborne MNPs to the Earth’s surface, where agricultural soils and terrestrial ecosystems become long-term accumulation sites, further complicating the food security landscape.

### 2.4. Current Status of Microplastic Exposure in the Soil

#### 2.4.1. Soil Detection Technology and Distribution Laws

Stereomicroscopy combined with FTIR spectroscopy remains the most common method for identifying soil microplastics. Advanced techniques like LDIR imaging, Pyr-GC–MS, and Raman spectroscopy are used less frequently [100]. Studies published between 2021 and 2025, sourced from the Web of Science database using the term “soil microplastic deposition,” provide insights into the global presence of microplastics (MPs) in agricultural land, gardens, orchards, forests, urban areas, marshes, and mudflats (Tables S2 and S3) [101,102].

#### 2.4.2. Effects of Land Use Types on Accumulation

In Chinese agricultural soils, MP levels differ considerably on the basis of location, crop type, soil morphology, and fertilization/irrigation practices [92,100]. Additionally, MPs’ abundance is consistently higher in surface soils (0–20 cm) compared with deeper layers, often by a factor of 3–5 [103]. Compacted soils, particularly those rich in clay, hinder MPs’ migration, leading to greater surface accumulation [104]. European studies indicate higher MP levels in farmlands fertilized with sewage sludge compared with untreated soils. Urban soils generally show greater contamination than rural soils, attributed to litter and industrial activity [105]. Similarly, sewage sludge application contributes an estimated 30,000 tons of MPs annually to North American agricultural soils [106]. Even remote areas like the Antarctic are affected, with fragment-shaped MPs found in terrestrial topsoil at concentrations of 1–37 particles per 50 mL of soil, suggesting contamination linked to human activity since the 1950s [107,108]. Notably, MPs’ persistence in the soil depends on the organic carbon content, iron/aluminum oxide concentrations, pH, and ionic strength [109].

A critical insight from this synthesis is the consistent vertical stratification of MPs across diverse geographical contexts, with surface layers (0–20 cm) holding 3–5 times higher loads than subsurface layers. This pattern establishes surface soil as both a sink and a potential source for food chain transfer, particularly for crops and soil biota. Despite progress, the lack of standardized methodologies for sampling, extraction, and analysis hinders comprehensive global assessments. Three major anthropogenic sources emerge from this evaluation: leachate infiltration from landfills, fragmentation of agricultural plastic films, and repeated application of sewage sludge fertilizers. These findings underscore the urgent need for unified protocols encompassing representative sampling strategies, optimized density separation techniques, and validated spectroscopic identification methods.

## 3. Adverse Effects of MNP Exposure on Organisms

Human exposure to micro(nano)plastics (MNPs) occurs inevitably via ingestion, inhalation, and dermal contact. MNPs have been detected in diverse human tissues, including the lungs [110], sputum [111], feces [112], and colon [113], but also within internal organs and protected sites, including the blood [114], placenta [17], testicles [18], and heart [115]. This pervasive distribution correlates with detrimental effects on multiple organ systems. The digestive and respiratory tracts serve as primary entry points, with subsequent distribution potentially affecting the circulatory, nervous, endocrine, and reproductive systems. This section synthesizes the current evidence, with a focus on elucidating shared and system-specific pathological mechanisms. A critical prerequisite for MNPs’ toxicity, especially for nanoplastics (NPs), is cellular internalization. NPs can enter cells via endocytic pathways such as clathrin- and caveolin-mediated endocytosis, influenced by their size, surface charge, and the acquired “eco-corona”. Once intracellular, MNPs can instigate a cascade of molecular events. Common mechanisms across organ systems include oxidative stress, inflammation, metabolic disruption, apoptosis, and cell death. Notably, although visually small, MNPs are physically the same polymers as macroplastics. Thermoplastic plastics (TPs) contain polymerization initiators, unreacted monomers, and up to 30 types of stabilizers that exist dispersed within the plastic matrix without chemically bonding to it. Furthermore, MNPs are predicted to have a larger surface area than macroplastics, which accelerates their degradation and increases the adsorption properties of persistent organic pollutants (POPs) and polychlorinated biphenyls (PCBs). Kinetically, it has been proven that TPs can decompose at low temperatures of 30–50 °C into their raw material monomers. Given that the human body temperature is approximately 36 °C, and marine mammals (e.g., whales, sea lions) maintain temperatures around 40 °C, TPs ingested into the body will inevitably decompose over time, exposing organisms not only to physical particle damage but also to sustained chemical toxicity from leached monomers.

### 3.1. Microplastic Risks to the Circulatory System

This review synthesizes recent experimental data elucidating cardiovascular pathologies in animal models (Table 3). MNP exposure induces multi-faceted cardiovascular toxicity. Key pathological outcomes include accelerated atherosclerosis, driven by MNP-induced endothelial dysfunction, macrophage activation (e.g., via MARCO receptors), lipid metabolism dysregulation, and vascular smooth muscle cell (VSMC) phenotypic transformation. MNPs also promote myocardial injury, including fibrosis (via the TGF-β/Smad pathway), hypertrophy, and inflammation. Clinical observations support these findings, with higher MNP loads detected in human atherosclerotic plaques and thrombi, and levels correlating with inflammatory markers in acute myocardial infarction patients. Limitation: Most evidence derives from high-dose PS-NP exposure in rodent models, which may not fully recapitulate chronic, low-dose human exposure to complex MNP mixtures [22].

#### 3.1.1. Inflammation and Immune Activation, Oxidative Stress, and Metabolic Dysregulation

Current research on the cardiovascular toxicity of microplastics primarily utilizes animal models, demonstrating that microplastics induce cardiovascular cellular inflammatory responses (elevated IL-6 and IL-12p70), immune activation (B-cell and NK-cell proliferation), oxidative stress, and lipid metabolism dysregulation, ultimately promoting atherosclerosis (AS) formation. However, most animal experiments have investigated acute high-dose exposure to polystyrene nanoplastics (PS-NPs), a paradigm markedly divergent from real-world human exposure, which typically involves chronic, low-dose exposure to complex polymer mixtures. Moreover, the lack of systematic comparative analyses of toxic effects across different polymer types limits the generalizability of the current findings.

#### 3.1.2. Atherosclerosis and Thrombosis

Evidence from the detection of microplastics within the human cardiovascular system indicates significantly higher concentrations in the coronary and carotid plaques of patients with atherosclerosis compared with normal aortic tissue [121,122]. Furthermore, irregular microplastic aggregates (2.1–26.0 μm) were identified in 16 out of 26 thrombus samples from cardiovascular surgery patients, suggesting a potential involvement in thrombotic processes [123]. Critically, the microplastic burden was significantly elevated in patients with acute myocardial infarction (AMI) versus those with unstable angina (UA), and total microplastic levels correlated positively with inflammatory cytokine concentrations (IL-6, IL-12p70), B cell populations (CD3^−^/CD19^+^), and natural killer (NK) cell populations (CD3^−^/CD56^+^/CD16^+^) [124].

### 3.2. Microplastic Risks to the Digestive System

#### 3.2.1. Intestinal Aggregation and Damage

Microplastic exposure in oysters causes accumulation within the gills and digestive glands, inducing histopathological damage and lipid metabolism inhibition [125]. In mice, polylactic acid (PLA) exposure disrupts the intestinal epithelial barrier, significantly inhibits growth, and enhances oxidative stress while causing intestinal flora dysbiosis [126]. Japanese quail exposed to polystyrene (PS) exhibit cecal damage, including microvillar injury, mitochondrial vacuolation, mucus layer rupture, reduced mucin secretion, immune dysfunction, and inflammatory responses [127].

#### 3.2.2. Hepatic Metabolic Disturbances and Fibrosis

Upon reaching the liver, MNPs disrupt cellular energy metabolism. Studies in zebrafish and mice show that MNPs, particularly polystyrene, can impair mitochondrial function, inhibit the citric acid cycle and oxidative phosphorylation, and lead to abnormalities in glucose and lipid metabolism, contributing to conditions like metabolism-associated fatty liver disease (MAFLD) [128,129,130,131,132].

Furthermore, MNPs activate inflammatory pathways, notably the NLRP3 inflammasome, triggering the release of pro-inflammatory factors (e.g., IL-1β) and promoting the progression from steatosis to hepatic fibrosis [133,134]. Co-exposure with other pollutants, such as heavy metals, can produce synergistic effects that accelerate fibrotic processes [26].

Microplastics are detected throughout the human digestive system, with variations in polymer type and particle size influencing site-specific deposition (Table 4). Critically, particle size governs their fate: larger microplastics are predominantly excreted via feces, whereas smaller particles exhibit greater potential for deposition within digestive tissues or translocation into the bloodstream, enabling accumulation in extraintestinal organs.

#### 3.2.3. Gut–Brain Axis (GBA)-Mediated Neurodegenerative Diseases

Intestinal microplastics induce physical damage to the mucosal barrier, triggering local inflammation and contributing to inflammatory bowel disease (IBD). Critically, they also alter the gut microbiota’s composition, disrupting host–microbiota symbiosis. This dysbiosis impairs microbial metabolite production and interferes with immune and neural regulatory functions. A novel contribution of this review is the synthesis of evidence linking microplastic-induced gut disruption to neurodegenerative diseases including Alzheimer’s disease (AD) and Parkinson’s disease (PD) through the gut–brain axis (GBA) [23]. This mechanistic pathway represents an emerging frontier in microplastic toxicology, with significant implications for chronic neurological disease risk.

### 3.3. Microplastics’ Risks to the Respiratory System

#### 3.3.1. Deposition, Clearance Impairment, and Direct Injury

Inhaled microplastics (MNPs) pose a direct risk to respiratory health, with effects depending on the particle size, polymer type, and co-exposures: particles > 10 μm primarily deposit in the upper airways via inertial impaction; particles < 10 μm reach the bronchioles; and particles < 2.5 μm deposit in the alveoli, with ultrafine fractions (<0.1 μm) potentially translocating into systemic circulation [139,140,141]. This deposition can cause physical damage (airway obstruction, epithelial abrasion) and impair mucociliary function, reducing clearance. MNPs can also act as carriers for adsorbed pathogens (e.g., *Streptococcus pneumoniae*), potentially exacerbating infections like community-acquired pneumonia.

#### 3.3.2. Key Molecular Mechanisms of Toxicity

Most contemporary studies utilize in vitro and animal models to investigate respiratory impacts. Firstly, regarding surfactant dysfunction and alveolar injury, polystyrene MNPs can disrupt the composition and function of lung surfactants, compromising alveolar stability even at low doses. In terms of oxidative stress and inflammation, MNPs induce reactive oxygen species (ROS), leading to oxidative damage. They can also activate inflammatory pathways (e.g., TLR4) and promote a pro-inflammatory shift in the lung microbiome [142]. They also activate the cGAS/STING pathway to trigger ferroptosis in alveolar epithelial cells, a key mechanism in the pathogenesis of idiopathic pulmonary fibrosis [143,144]. Notably, multiple microplastic polymers (PE, PP, PS, PVC) induce cellular senescence in human lung epithelial cells via ROS-mediated redox imbalance [145]. Furthermore, inhaled MPs (especially fragments/fibers < 10 μm) damage ciliated epithelia, impair mucociliary clearance, adsorb pathogens (e.g., *Streptococcus pneumoniae*), and potentiate community-acquired pneumonia through synergistic effects [146].

#### 3.3.3. Potential Risk of Lung Cancer

Clinical evidence suggests microplastics’ involvement in pulmonary carcinogenesis. For instances, allergic rhinitis patients exhibit significantly elevated nasal microplastic loads versus controls, indicating chronic inflammation linkage [147]. MNPs’ detection rates are higher in lung carcinoma tissue (particularly ground-glass nodule regions) than in normal parenchyma [148]. Moreover, in vivo models demonstrate that aged polypropylene MNPs amplify lung cancer risk through physical tissue damage, heavy metal-microplastic composite (MMC) formation, oxidative stress amplification, oncogenic pathway activation (e.g., MAPK, NF-κB), and tumor microenvironment remodeling [149].

### 3.4. Microplastic Risks to Endocrine and Reproductive Systems

#### 3.4.1. Endocrine Disruption

The endocrine-disrupting effects of MNPs are primarily driven by the chemical components (monomers, additives) released as thermoplastics degrade in vivo. Key substances include bisphenol A (BPA) and phthalate esters, which are established endocrine-disrupting chemicals (EDCs).

In males, components like styrene oxides from polystyrene can accumulate in the testes, inducing oxidative stress (e.g., via NOX4 activation) and inhibiting key antioxidant enzymes (e.g., GPX1). The resulting peroxide accumulation directly suppresses steroidogenic enzymes, ultimately blocking testosterone synthesis [150].

**Figure 4:**
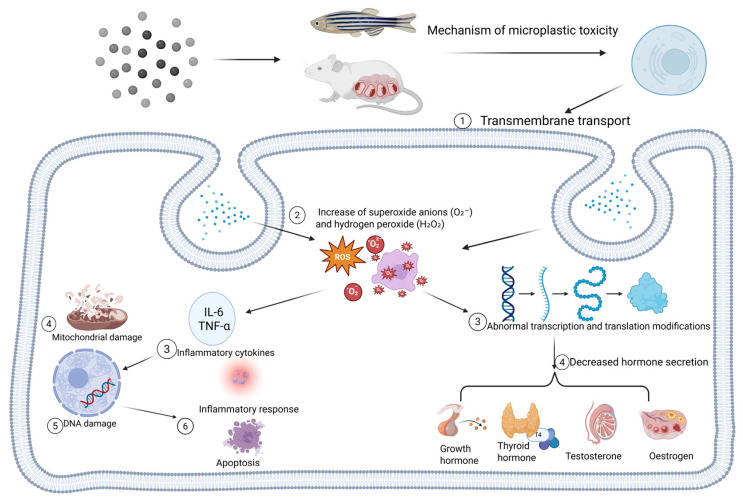
Mechanism of endocrine toxicity of microplastics.

In females, MNPs disrupt ovarian function by directly inhibiting estrogen synthesis, promoting granulosa cell apoptosis, and causing dysregulation of the hypothalamic–pituitary–ovarian (HPO) axis. As summarized in Figure 4, this initiates a self-perpetuating cycle of oxidative stress, apoptosis, and inflammation that impairs overall reproductive endocrine homeostasis [151].

#### 3.4.2. Transgenerational Toxicity

MNPs have been detected in the human placenta at concentrations ranging from 6.5 to 685 μg/g, confirming their ability to cross the placental barrier [152]. A significant contribution of this review is the synthesis of emerging evidence on transgenerational toxicity, a critical knowledge gap in prior syntheses. Animal studies demonstrate that maternal exposure to polyethylene microplastics (PE-MPs) induces offspring abnormalities, including reduced live birth rates, imbalanced sex ratios, and altered body weight, as well as alterations in splenic lymphocyte subpopulations [153]. Exposure to polystyrene microplastics (PS-MPs) during pregnancy and lactation disrupts cortical neurotransmitter levels in fetal rats, leading to cortical hyperproliferation, hippocampal synaptic damage, and consequent spatial memory deficits and anxiety-like behaviors in the offspring [154]. The primary mechanisms underlying transgenerational MP toxicity likely include oxidative stress and epigenetic alterations [155], endocrine axis disruption [150], and signal pathway dysfunction [156,157,158].

#### 3.4.3. Thyroid and Growth Hormone Axis Dysfunction

Thyroid Hormone Synthesis Disruption: Exposure to polystyrene nanoplastics (PS-NPs) impairs iodine uptake and utilization in rodent models. This occurs through direct physical interference with the sodium iodide symporter (NIS) and key enzymes (thyroid peroxidase, deiodinases), as well as epigenetic suppression of thyroglobulin synthesis. Concurrent oxidative stress and inflammation (via NF-κB) further contribute to thyroid tissue damage [25].

Parathyroid Hormone and Calcium Dysregulation: PS-NPs also disrupt parathyroid function, inhibiting the receptors and gene expression critical for maintaining calcium homeostasis and leading to abnormal parathyroid hormone secretion [159]. 

Suppression of the GH/IGF-1 Axis and Axis Cross-talk: The presence of MPs activates inflammatory pathways (TLR4/NF-κB) and induces chronic oxidative stress, which collectively suppress the secretion of growth hormone-releasing hormone (GHRH) and growth hormone (GH). Additionally, the low thyroid hormone (T3) state induced by MPs reduces hepatic GH receptors’ sensitivity, creating a self-perpetuating “GH resistance–low IGF-1” cycle. This IGF-1 deficiency further exacerbates peripheral T3 deficiency, demonstrating a critical vicious cycle between the disrupted GH/IGF-1 and hypothalamic–pituitary–thyroid (HPT) axis [160,161].

## 4. Summarizing and Perspectives

### 4.1. Current Challenges

MNP research faces several critical challenges beyond the previously noted limitations. Despite recent advances, translating mechanistic insights regarding micro- and nanoplastics (MNPs) into robust human health risk assessments is impeded by several persistent methodological and knowledge gaps. Fundamentally, the absence of standardized sampling protocols precludes meaningful cross-comparisons between active and passive monitoring datasets. This methodological limitation is compounded by the complex environmental behavior of MNPs; for instance, the specific mechanisms governing concentration gradients across disparate terrestrial environments—spanning urban, rural, coastal, and inland soils—require further elucidation. This complexity extends to the marine sink, where the recent quantification of toxic, metabolizable monomeric transformation products (TPs) at abyssal depths (2000–5000 m) severely complicates global exposure models. From a biological and toxicological perspective, the dynamic in vivo degradation of MNPs and their precise environmental fate remain poorly characterized. Specifically, the degradation kinetics of TPs at physiological temperatures (36–40 °C) constitutes a critical blind spot in current research. Furthermore, demographic variables, such as the exact factors driving differential inhalation patterns between pediatric and adult populations, are not yet fully resolved. Ultimately, elucidating the synergistic toxicities of composite pollutants—such as polypropylene–cadmium (PP-Cd^2+^) complexes and co-accumulated persistent organic pollutants (POPs/PCBs)—alongside biofilm-mediated transport mechanisms, is imperative for advancing predictive toxicology and environmental health frameworks.

### 4.2. Future Advancements and Directions

Future work must address these challenges. A critical first step is the development of globally recognized protocols—such as ISO standards for atmospheric MNP quantification—alongside the construction of age-stratified inhalation models to yield higher-fidelity risk assessments. Furthermore, resolving the complex physicochemical behavior of MNPs dictates the integration of advanced, hyphenated analytical techniques. For instance, coupling laser direct infrared (LDIR) imaging with Raman spectroscopy offers a robust platform for comprehensive detection, while in situ modalities like cryo-electron microscopy (cryo-EM) are indispensable for characterizing “environmental corona” formation and the interfacial interactions between MNPs and co-contaminants. Ultimately, toxicological investigations must transition toward environmentally realistic paradigms, specifically focusing on chronic, low-dose exposures to aged MNP mixtures. Within this context, elucidating the specific pathways of MNP-induced neurotoxicity via the gut–brain axis, as well as the epigenetic mechanisms underpinning transgenerational effects, should emerge as central research priorities.

### 4.3. Policy Discussion

This review calls for targeted policy actions built on three pillars. First, standardization is imperative, requiring the development of ISO/ASTM protocols for microplastic detection and an international toxicity classification system. Second, pollution control must be prioritized through mandates on biodegradable agricultural films, industrial wastewater filtration, and maritime emission limits. Third, risk-based governance should be implemented, featuring differentiated strategies for zone-specific remediation, soil rehabilitation, and protection of vulnerable populations via ventilation guidelines and dietary intake standards. Critically, this risk-based governance must be expanded to regulate the chemical complexity of thermoplastics (TPs). Policies must strictly control the use of unbonded stabilizers, polymerization initiators, and unreacted monomers in plastic manufacturing to prevent the leaching of carcinogenic and endocrine-disrupting chemicals (e.g., BPA, PAE, SOs). These measures form the cornerstone of a comprehensive strategy to address microplastic exposure and its associated health risks.

### 4.4. Conclusions

Micro- and nanoplastics (MNPs) are now recognized as pervasive environmental contaminants across marine, atmospheric, and terrestrial ecosystems, resulting in chronic, multi-route human exposure via ingestion, inhalation, and dermal contact. The profound physicochemical heterogeneity of MNPs—encompassing diverse polymers, morphometries, and their capacity to act as vectors for co-contaminants (the “Trojan horse” effect)—complicates their toxicological profiling compared with traditional chemical pollutants. As synthesized in this review, MNPs demonstrate the capacity to translocate across critical biological interfaces, notably the intestinal epithelium, blood–brain barrier, and placenta. This translocation induces localized inflammation, oxidative stress, and metabolic perturbation, ultimately driving the cardiovascular, gastrointestinal, respiratory, and endocrine/reproductive toxicities detailed in Section 3.1, Section 3.2, Section 3.3 and Section 3.4. Furthermore, the potential for MNP-induced transgenerational toxicity and the disruption of neuroendocrine–immune axes warrant urgent attention, given their possible contribution to the global etiology of chronic inflammatory conditions. Despite these findings, robust human health risk assessments remain constrained by significant methodological limitations. Primarily, the absence of standardized analytical protocols for quantifying nanoscale plastics within complex biological matrices impedes effective clinical biomonitoring. Additionally, the prevalent reliance on high-dose, pristine spherical models fails to recapitulate the chronic toxicity of environmentally weathered, morphologically diverse plastic debris. To bridge the translational gap between experimental toxicology and epidemiological outcomes, future research must adopt environmentally realistic exposure paradigms. Ultimately, mitigating MNP-related health risks necessitates the integration of harmonized biomonitoring frameworks, rigorous mechanistic toxicology, and evidence-based global policy interventions.

## Figures and Tables

**Table 1:** Abundance and exposure risk assessment of microplastics in different regions.

Region	Abundance	Polymer Type	PHI/H	PLI	References
1. Surface waters off Guangdong Province	295.3 ± 175.3 items/m^3^	PP, PE	PHI_average_: 223.76–622.0 (III)	1.51–2.4 (II)	[41]
2. Surface water of Qinhuai River in Nanjing	466.62 ± 153.69 items/L	PVC, PET, PC	PHI_average_: (IV)	(I)	[42]
3. Surface water of lakes in the inner city of Da Nang, Vietnam	Rainy season: 643.8 ± 87.5 items/m^3^	PP, PE, PET	PHI_PP_: (I) PHI_PE,PET_: (II)	2.05–9.64 (I) Rainy season: 2.29–4.04 (I)	[43]
4. Surface waters of St. Mary’s Island, India	0.218 ± 0.329 items/L	LDPE, PS, PA, PP, PE	PHI_PP_: 11.8 (I), PHI_PS_: 847.3 (III)	(I)	[44]
5. Surface waters of the Lhasa River, Tibetan Plateau	0.63 items/L	PP, PE, PVC	PHI_average_: (III)	(I)	[45]
6. Surface water in Antarctica	0–0.56 items/m^3^	PVC	PHI_average_: (IV)	3.14 (I)	[46]
7. Coastal sediments of Haizhou Bay, Lianyungang, China	1.01 ± 1.28 items/g	PET	PHI_PA,PET_: (II)	1.95 (I)	[47]
8. Estuarine sediments in Liaodong Bay waters	32.33–49.91 items/kg	PET, PA, PP, PE	/	(I)	[48]
9. Beach sediments of Gulf of Mannar, India	33.82 ± 26.11 items/kg	PE	PHI_average_: 698.96 (IV)	2.51 (I)	[49]
10. Sediments of the Karnaphuli estuary, Bangladesh	22.29–59.5 items/kg	PE, PS, PET, Nylon, PVC	PHI_average_: (IV)	1.73 (I)	[50]
11. Caribbean coast of Colombia	102 ± 7.86 items/kg	PP, PE, PS	PHI_average_: 2.0–16.7 (II)	(I)	[51]
12. Limfjord Northern Denmark	1863 ± 1163 items/kg	PP, PE, PS, PAN	PHI_average_: 132.8 (III)	29.6 (IV)	[52]
13. Southwestern Atlantic coast of Argentina	12.8 ± 3.2 items/kg	PVC, PP, PET	/	3.8 (I)	[53]

Abbreviations: PHI/H: Polymer Hazard Index; PE: polyethylene; LDPE: low-density polyethylene; PVC: polyvinyl chloride; PET: polyethylene terephthalate; PC: polycarbonate; PS: polystyrene; PA: polyamide; PP: polypropylene; PAN: polyacrylonitrile; PLI method: Class I: low pollution load; Class II: medium pollution load; Class III–V: high to very high pollution loads. HI Risk Rating: low risk (Class I to II); medium risk (Class III); high risk (Class IV).

**Table 2:** Concentrations of MPs measured indoors and outdoors in different regions.

Location	Type	Concentration	Polymer Type	Identification Methods	References
Indoor					
Paris, France	Uptown	5.4 particles/m^3^	PE, PP, PS	PSM	[72]
Queensland, Australia	Uptown	0.20~2.25 particles/m^3^	PET, PE	PSM	[73]
Yorkshire–Humber	Uptown	1414 ± 1022 particles/m^2^/d	PET, PA, PP	PASM	[74]
		4.8 ± 1.6 particles/m^3^	PE, PP, PE	PSM	[59]
		0.13~0.93 particles/m^3^	PET	PASM	[60]
		0.148–0.31 particles/m^3^	PE, PP, PS	PSM	[61]
		0.6–1.3 particles/m^3^	/	PSM	[62]
		1583 ± 1181 particles/m^3^	PE, PS	PSM	[63]
Barcelona, Spain	Uptown	4.8 ± 1.6 particles/m^3^	PE, PP, PS	PSM	[59]
Sri Lanka region					
Mexico City					
Aveiro, Portugal					
Wenzhou, Zhejiang					
Sri Lanka region	Uptown	0.13~0.93 particles/m^3^	PET, PE	PSM	[60]
Mexico City	Uptown	0.148–0.31 particles/m^3^	PET, PA, PP	PSM	[61]
		4.8 ± 1.6 particles/m^3^	PE, PP, PE	PSM	[59]
		0.13~0.93 particles/m^3^	PET	PASM	[60]
		0.148–0.31 particles/m^3^	PE, PP, PS	PSM	[61]
		0.6–1.3 particles/m^3^	/	PSM	[62]
		1583 ± 1181 particles/m^3^	PE, PS	PSM	[63]
Wenzhou, China	Uptown	1583 ± 1181 particles/m^3^	PET, PE	PSM	[74]
Outdoor					
Wenzhou, China	Urban	224 ± 70 particles/m^3^	PE, PS, PET	PSM	[74]
	Rural	101 ± 47 particles/m^3^	/	PSM	
Shanghai, China	Urban	72–144 particles/m^3^	PET, PT, PES	PSM	[75]
Tempe, Arizona	Urban	0.02–1.1 particles/m^3^	/	PSM	[76]
Handan, China	Rural	7301 particles/m^2^/d	PET, PE	PASM	[77]
Nanjing, China	Urban	143.3 ± 40.8 particles/m^3^	PS, PA	PSM	[78]
Nagpur, India	Urban	116.25 ± 26.4 particles/m^2^/d	PE, PP	PASM	[79]
Manila, Philippines	Urban	0.021 ± 0.006 particles/m^3^	PET, PA	PSM	[80]
Tibetan Plateau, China	Remote area	2.5–58.8 particles/m^3^	PET, PE, PA,	PSM	[81]
Foothills of Western Alps, New Zealand	Remote area	150 particles/m^2^/d	PS, PET PA, PET	PASM	[82]
Plymouth, UK	Urban	0.082 ± 0.06 particles/m^3^	PA, PET	PSM	[83]
Mount Derak, Iran	Remote area	0.51 ± 0.20 particles/m^3^	/	PSM	[84]
Shiraz Iran	Urban	2.65 ± 1.44 particles/m^3^			
Paris, France	Urban	0.3–1.5 particles/m^3^	/	PSM	[72]

Abbreviations. PE: polyethylene; PET: polyethylene terephthalate; PS: polystyrene; PP: polypropylene; PA: polyamide. PSM: plastic source material; PASM: plastic additive source material.

**Table 3:** Studies of the effects of microplastic exposure on cardiovascular system damage in animal models.

Animal Model	Treatment with PS-NPs	Observations	References
Apo E^−/−^ male mice	25, 250 mg/kg PS-NPs via gavage with high-fat diet	PS-NP exposure accelerated atherosclerotic plaque progression in mice, linked to upregulated MARCO expression in macrophages which caused dyslipidemia and disrupted lipid metabolism	[22]
Apo E^−/−^ male mice	2.5, 25, 250 mg/kg PS-NPs via gavage with high-fat diet	The exposed group showed increased plaque area and foam cell numbers within plaques, triggering an aortic inflammatory microenvironment and promoting vascular smooth muscle cell (VSMC) migration into plaques.	[116]
Wistar male rats	0.5, 5, 50 mg/kg/d PS-NPs administered via gavage for 90 days	PS-NP exposure disordered vascular endothelial cell arrangement, induced inflammatory cell infiltration, and increased expression of GSDMD and the NLRP3 inflammasome-mediated pyroptosis pathway.	[117]
C57BL/6 male mice	10–100 μg/mL PS-NPs applied in cell culture media	Moderate PS-NP exposure induced phenotypic transformation in damaged VSMCs, while high concentrations caused apoptosis, severe mitochondrial damage (ROS overproduction, mutant mtDNA accumulation), and dysregulated mitochondrial dynamics genes, alongside tiRNA-Glu-CTC overexpression, promoting vascular injury.	[118]
SPF BALB/c male mice	0.025, 0.25, 2.5 μg/mL PS-NPs via intratracheal drip twice weekly	Exposure to MP-NPs in mice and cardiac organoids induced mild oxidative stress, increased inflammation, cell death, organoid volume, collagen accumulation, and disordered cell arrangement, along with significant upregulation of myocardial hypertrophy markers (MYH7B, ANP, BNP, COL1A1).	[119]
Mouse monocyte-macrophage, adult zebrafish	0.1 to 1.5 mg/mL PS-NPs applied to cells 0.5 mg/mL PS-NPs introduced into the water environment	PS-NP exposure significantly reduced macrophages’ viability, increased apoptosis, and decreased cell numbers in vivo, while also altering the macrophage metabolic profile, reducing sphingolipid metabolism specificity and causing abnormal lipid metabolism.	[120]

**Table 4:** Microplastic accumulation in organs of the human digestive system.

Sample	Sources	Particle Size	Polymer Type	Microplastic Abundance	References
Feces	26 male university students	20–800 μm	PP, PET, PS, PE, PA, PC, PVC	1–36 particles/g	[135]
Feces	6 infants and toddlers (1 years) 3 newborns 10 Adults	/	PET, PC	Infants: 5.756–84.1 μg/g; newborns: 0–12 μg/g; adults: 0.093–16.13 μg/g	[136]
Tonsil	/	20–200 μm	PVC	6.03 particles/g	[137]
Intestine	/	20–200 μm	PVC, PS, PE	9.45 particles/g	[137]
Colon	6 men and 5 women	800–1600 μm	PP, PC, PA	28.1 ± 15.4 particles/g	[113]
Liver	/	5–30 μm	PS, PVC, PET, PMMA, PP	0–1.5 particles/g	[138]

Abbreviations. PE: polyethylene; PET: polyethylene terephthalate; PS: polystyrene; PC: polycarbonate; PP: polypropylene; PA: polyamide; PVC: polyvinyl chloride; PMMA: poly methyl methacrylate.

## Data Availability

No new data were created or analyzed in this study. Data sharing is not applicable to this article.

## Supplementary Materials

The following supporting information can be downloaded at https://www.mdpi.com/article/10.3390/toxics14050442/s1, Figure S1: Proportion and Production of plastic products in different regions around the world from 2020 to 2023; Figure S2. Average microplastic exposure doses for different age groups outdoors in different regions (MPs/kg-BW/d); Figure S3. Average microplastic exposure doses for different age groups indoors in different regions (MPs/kg-BW/d); Figure S4. Average microplastic exposure doses for different age groups outdoors of different regions in China (MPs/kg-BW/d); Table S1: Risk Level Categories of Microplastics; Table S2. Status of soil microplastics in China; Table S3. Status of soil microplastics in Europe.

## References

[1] Geyer R., Jambeck J.R., Law K.L. (2017). Production, Use, and Fate of All Plastics Ever Made. Sci. Adv..

[2] Haider T.P., Voelker C., Kramm J., Landfester K., Wurm F.R. (2019). Plastics of the Future? The Impact of Biodegradable Polymers on the Environment and on Society. Angew. Chem. Int. Ed..

[3] Issifu I., Dahmouni I., Sumaila U.R. (2025). Assessing the Ecological and Economic Transformation Pathways of Plastic Production System. J. Environ. Manag..

[4] Thompson R.C., Olsen Y., Mitchell R.P., Davis A., Rowland S.J., John A., McGonigle D., Russell A.E. (2004). Lost at Sea: Where is All the Plastic?. Science.

[5] Bibas R., Chateau J., Lanzi E., Mavroedi E., Valribéras D.O. Global Plastic Projections to 2050: Economic Drivers and Environmental Consequences 2020. https://ageconsearch.umn.edu/record/333217/files/9685.pdf.

[6] Landrigan P., Symeonides C., Raps H., Dunlop S. (2023). The global plastics treaty: Why is it needed?. Lancet.

[7] Markic A., Niemand C., Bridson J.H., Mazouni-Gaertner N., Gaertner J.C., Eriksen M., Bowen M. (2018). Double trouble in the South Pacific subtropical gyre: Increased plastic ingestion by fish in the oceanic accumulation zone. Mar. Pollut. Bull..

[8] Gan Q., Cui J., Jin B. (2023). Environmental microplastics: Classification, sources, fates, and effects on plants. Chemosphere.

[9] Huang W., Xia X. (2024). Element cycling with micro(nano)plastics. Science.

[10] Wang T., Wang L., Chen Q., Kalogerakis N., Ji R., Ma Y. (2020). Interactions between microplastics and organic pollutants: Effects on toxicity, bioaccumulation, degradation, and transport. Sci. Total Environ..

[11] Zhang Y., Kang S., Allen S., Allen D., Gao T., Sillanpää M. (2020). Atmospheric Microplastics: A Review on Current Status and Perspectives. Earth-Sci. Rev..

[12] Andrady A.L. (2011). Microplastics in the marine environment. Mar. Pollut. Bull..

[13] Ramsperger A.F.R.M., Bergamaschi E., Panizzolo M., Fenoglio I., Barbero F., Peters R., Undas A., Purker S., Giese B., Lalyer C.R. (2023). Nano- and Microplastics: A Comprehensive Review on Their Exposure Routes, Translocation, and Fate in Humans. NanoImpact.

[14] Sangkham S., Faikhaw O., Munkong N., Sakunkoo P., Arunlertaree C., Chavali M., Mousazadeh M., Tiwari A. (2022). A Review on Microplastics and Nanoplastics in the Environment: Their Occurrence, Exposure Routes, Toxic Studies, and Potential Effects on Human Health. Mar. Pollut. Bull..

[15] Leslie H.A., van Velzen M.J.M., Brandsma S.H., Vethaak A.D., Garcia-Vallejo J.J., Lamoree M.H. (2022). Discovery and quantification of plastic particle pollution in human blood. Environ. Int..

[16] Singh S., Jain M.S. (2026). Terrestrial Microplastics as Emerging Aquatic Pollutants: A Systematic Review. J. Hazard. Mater. Plast..

[17] Zhu L., Zhu J., Zuo R., Xu Q., Qian Y., An L. (2023). Identification of Microplastics in Human Placenta Using Laser Direct Infrared Spectroscopy. Sci. Total Environ..

[18] Zhao Q., Zhu L., Weng J., Jin Z., Cao Y., Jiang H., Zhang Z. (2023). Detection and characterization of microplastics in the human testis and semen. Sci. Total Environ..

[19] Roman J., Gondko D., Biec P.D., Pietrzak N. (2024). The Hidden Health Crisis: Microplastics and Their Medical Consequences. J. Educ. Health Sport.

[20] Nolan T. (2025). Microplastics in Brains … and Other Research. BMJ.

[21] Amato-Lourenco L.F., Dantas K.C., Junior G.R., Paes V.R., Ando R.A., de Oliveira Freitas R., Costa O.M.M.M., Rabelo R.S., Bispo K.C.S., Carvalho-Oliveira R. (2024). Microplastics in the Olfactory Bulb of the Human Brain. JAMA Netw. Open.

[22] Wang B., Liang B., Huang Y., Li Z., Zhang B., Du J., Ye R., Xian H., Deng Y., Xiu J. (2023). Long-Chain Acyl Carnitines Aggravate Polystyrene Nanoplastics-Induced Atherosclerosis by Upregulating MARCO. Adv. Sci..

[23] Sofield C.E., Anderton R.S., Gorecki A.M. (2024). Mind over Microplastics: Exploring Microplastic-Induced Gut Disruption and Gut-Brain-Axis Consequences. Curr. Issues Mol. Biol..

[24] Qu J., Wu L., Mou L., Liu C. (2024). Polystyrene microplastics trigger testosterone decline via GPX1. Sci. Total Environ..

[25] Zhang J., Liu L., Dai X., Li B., Zhang S., Yu Y. (2024). Thyroid and parathyroid function disorders induced by short-term exposure of microplastics and nanoplastics: Exploration of toxic mechanisms and early warning biomarkers. J. Hazard. Mater..

[26] Sun J., Qu H., Ali W., Chen Y., Wang T., Ma Y., Yuan Y., Gu J., Bian J., Liu Z. (2023). Co-exposure to Cadmium and Microplastics Promotes Liver Fibrosis Through the Hemichannels-ATP-P2X7 Pathway. Chemosphere.

[27] Kozlov M. (2024). Landmark study links microplastics to serious health problems. Nature.

[28] Bergmann M., Muetzel S., Primpke S., Tekman M.B., Trachsel J., Gerdts G. (2019). White and Wonderful? Microplastics Prevail in Snow from the Alps to the Arctic. Sci. Adv..

[29] Allen S., Allen D., Karbalaei S., Maselli V., Walker T.R. (2022). Micro(nano)plastics Sources, Fate, and Effects: What We Know after Ten Years of Research. J. Hazard. Mater. Adv..

[30] PlasticsEurope (2024). Plastics—The Fast Facts 2024. https://plasticseurope.org/knowledge-hub/plastics-the-fast-facts-2024/.

[31] Ma Y., Xie Z., Hamid N., Tang Q., Deng J., Luo L., Pei D. (2023). Recent Advances in Micro (nano) Plastics in the Environment: Distribution, Health Risks, Challenges and Future Prospects. Aquat. Toxicol..

[32] Singh T. (2021). Generation of microplastics from the opening and closing of disposable plastic water bottles. J. Water Health.

[33] Anagnosti L., Varvaresou A., Pavlou P., Protopapa E., Carayanni V. (2021). Worldwide actions against plastic pollution from microbeads and microplastics in cosmetics focusing on European policies. Has the issue been handled effectively?. Mar. Pollut. Bull..

[34] Gong J., Xie P. (2020). Research progress in sources, analytical methods, eco-environmental effects, and control measures of microplastics. Chemosphere.

[35] Vibhatabandhu P., Srithongouthai S. (2022). Abundance and Characteristics of Microplastics Contaminating the Surface Water of the Inner Gulf of Thailand. Water Air Soil Pollut..

[36] Suaria G., Perold V., Lee J.R., Lebouard F., Aliani S., Ryan P.G. (2020). Floating macro-and microplastics around the Southern Ocean: Results from the Antarctic Circumnavigation Expedition. Environ. Int..

[37] Dris R., Gasperi J., Saad M., Mirande C., Tassin B. (2016). Synthetic fibers in atmospheric fallout: A source of microplastics in the environment?. Mar. Pollut. Bull..

[38] Faure F., Demars C., Wieser O., Kunz M., De Alencastro L.F. (2015). Plastic pollution in Swiss surface waters: Nature and concentrations, interaction with pollutants. Environ. Chem..

[39] Tomlinson D.L., Wilson J.G., Harris C.R., Jeffrey D.W. (1980). Problems in the assessment of heavy-metal levels in estuaries and the formation of a pollution index. Helgoländer Meeresunters..

[40] Hakanson L. (1980). An ecological risk index for aquatic pollution control. A sedimentological approach. Water Res..

[41] Liu B., Ye K., Lu Y., Deng H., Yang J., Li K., Liu L., Zheng H., Sun K., Jiang Y. (2024). Occurrence and Risk Assessment of Microplastics in the Coastal Seawater of Guangdong Province. Huan Jing Ke Xue.

[42] Wang L., Huang J., Wu Y., Chen X., Chen M., Jin H., Yao J., Wang X. (2024). Spatial-Temporal and Risk Assessment of Microplastics in the Surface Water of the Qinhuai River during Different Rainfall Seasons in Nanjing City, China. Water.

[43] Tran-Nguyen Q.A., Le T.M., Nguyen H.N.Y., Nguyen Q.T., Trinh-Dang M. (2024). Microplastics in the surface water of urban lakes in central Vietnam: Pollution level, characteristics, and ecological risk assessment. Case Stud. Chem. Environ. Eng..

[44] Khaleel R., Valsan G., Rangel-Buitrago N., Warrier A.K. (2023). Microplastics in the marine environment of St. Mary’s Island: Implications for human health and conservation. Environ. Monit. Assess..

[45] Zhou A., Zhao Y., Liu M., Suyamud B., Yuan W., Yang Y. (2023). Occurrence and risk assessment of microplastics in the Lhasa River—A remote plateau river on the Qinghai-Tibet Plateau, China. Environ. Monit. Assess..

[46] Gurumoorthi K., Luis A.J. (2023). Recent trends on microplastics abundance and risk assessment in coastal Antarctica: Regional meta-analysis. Environ. Pollut..

[47] Gao C., Liang B., Zhang S. (2024). Accumulation characteristics and ecological risk evaluation of microplastics in sediment cores from the artificial reef area and surrounding seas of Haizhou Bay, north China. Sci. Total Environ..

[48] Ye Y., Zhang A., Teng J., Yang X., Yuan X., Wang Q., Zhao J., Zhang B., Zhang T., Chen X. (2023). Pollution characteristics and ecological risk of microplastic in sediments of Liaodong Bay from the northern Bohai Sea in China. Mar. Pollut. Bull..

[49] Rakib M.R.J., Hossain M.B., Kumar R., Ullah M.A., Al Nahian S., Rima N.N., Choudhury T.R., Liba S.I., Yu J., Khandaker M.U. (2022). Spatial distribution and risk assessments due to the microplastics pollution in sediments of Karnaphuli River Estuary, Bangladesh. Sci. Rep..

[50] Keerthika K., Padmavathy P., Rani V., Jeyashakila R., Aanand S., Kutty R. (2022). Contamination of microplastics, surface morphology and risk assessment in beaches along the Thoothukudi coast Gulf of Mannar region. Environ. Sci. Pollut. Res. Int..

[51] Fuentes Molina N., López Pérez T.M., Puerta Cerpa Y.D. (2025). Molecular mechanisms of microplastic toxicity in coastal sediments of La Guajira Colombia and emerging ecological risks. Case Stud. Chem. Environ. Eng..

[52] Simon-Sánchez L., Vianello A., Kirstein I.V., Molazadeh M.-S., Lorenz C., Vollertsen J. (2024). Assessment of microplastic pollution and polymer risk in the sediment compartment of the Limfjord, Denmark. Sci. Total Environ..

[53] Forero-López A.D., Toniolo M.A., Colombo C.V., Rimondino G.N., Cuadrado D., Perillo G.M.E., Malanca F.E. (2024). Marine microdebris pollution in sediments from three environmental coastal areas in the southwestern Argentine Atlantic. Sci. Total Environ..

[54] Lin V.S. (2016). Research highlights: Impacts of microplastics on plankton. Environ. Sci. Process. Impacts.

[55] Ugwu K., Herrera A., Gómez M. (2021). Microplastics in marine biota: A review. Mar. Pollut. Bull..

[56] Schwabl P., Köppel S., Königshofer P., Bucsics T., Trauner M., Reiberger T., Liebmann B. (2019). Detection of Various Microplastics in Human Stool. Ann. Intern. Med..

[57] Gunaalan K., Nielsen T.G., Torres R.R., Lorenz C., Vianello A., Andersen C.A., Vollertsen J., Almeda R. (2023). Is Zooplankton an Entry Point of Microplastics into the Marine Food Web?. Environ. Sci. Technol..

[58] Desforges J.-P.W., Galbraith M., Ross P.S. (2015). Ingestion of Microplastics by Zooplankton in the Northeast Pacific Ocean. Arch. Environ. Contam. Toxicol..

[59] De Pascalis F., De Felice B., Parolini M., Pisu D., Pala D., Antonioli D., Perin E., Gianotti V., Ilahiane L., Masoero G. (2022). The hidden cost of following currents: Microplastic ingestion in a planktivorous seabird. Mar. Pollut. Bull..

[60] Kalaiselvan K., Pandurangan P., Velu R., Robinson J. (2022). Occurrence of microplastics in gastrointestinal tracts of planktivorous fish from the Thoothukudi region. Environ. Sci. Pollut. Res..

[61] Jiang R., Deng Z., Li J., Xiao Y., Xu Y., Wang J., Li T., Zhang C. (2023). The “Journey” of Microplastics across the Marine Food Web in China’s Largest Fishing Ground. Water.

[62] Wardrop P., Shimeta J., Nugegoda D., Morrison P.D., Miranda A., Tang M., Clarke B.O. (2016). Chemical Pollutants Sorbed to Ingested Microbeads from Personal Care Products Accumulate in Fish. Environ. Sci. Technol..

[63] Yang H., Chen G., Wang J. (2021). Microplastics in the Marine Environment: Sources, Fates, Impacts and Microbial Degradation. Toxics.

[64] Ziccardi L.M., Edgington A., Hentz K., Kulacki K.J., Driscoll S.K. (2016). Microplastics as vectors for bioaccumulation of hydrophobic organic chemicals in the marine environment: A state-of-the-science review. Environ. Toxicol. Chem..

[65] Ashton K., Holmes L.A., Turner A. (2010). Association of metals with plastic production pellets in the marine environment. Mar. Pollut. Bull..

[66] Keswani A., Oliver D.M., Gutierrez T., Quilliam R.S. (2016). Microbial hitchhikers on marine plastic debris: Human exposure risks at bathing waters and beach environments. Mar. Environ. Res..

[67] Yu H., Li G., Zhang H., Li Q., Wang L., Zhang D. (2025). Adsorption mechanism of arsenic(V) on aged polyethylene microplastics: Isotherms, kinetics and effect of environmental factors. J. Hazard. Mater. Adv..

[68] Feng F., Wang S., He X., Wang X., Huang J., Liu G., Rong S., Su S., Yan H., Han B. (2025). Adsorption behavior and mechanism of cadmium, copper, and lead on polylactic acid microplastics exposed to ultraviolet light. J. Environ. Chem. Eng..

[69] Cheng X., Yang Z., Ji K., Hu Z., Xi Y., Xiang X. (2025). Enhanced copper adsorption by polyamide and polylactic acid microplastics: The role of biofilm development and chemical aging. Environ. Res..

[70] Wang T., Wang L., Chen Q., Kalogerakis N., Ji R., Ma Y. (2024). Interaction between microplastics and other environmental pollutants and the combined effects of the combined pollutants. Chin. J. Mar. Environ. Sci..

[71] Yang H., Yang J., Sun L., Mi Y., Yan H., Zou X., Wang C., Zang H., Cheng Y., Li C. (2025). The alteration in adsorption mechanism and associated bioaccessibility of mesotrione on virgin and aged biodegradable mulch film-derived microplastics. Chem. Eng. J..

[72] Dris R., Gasperi J., Mirande C., Mandin C., Guerrouache M., Langlois V., Tassin B. (2017). A first overview of textile fibers, including microplastics, in indoor and outdoor environments. Environ. Pollut..

[73] Perera K., Ziajahromi S., Nash S.B., Leusch F.D.L. (2023). Microplastics in Australian indoor air: Abundance, characteristics, and implications for human exposure. Sci. Total Environ..

[74] Liao Z., Ji X., Ma Y., Lv B., Huang W., Zhu X., Fang M., Wang Q., Wang X., Dahlgren R. (2021). Airborne microplastics in indoor and outdoor environments of a coastal city in Eastern China. J. Hazard. Mater..

[75] Liu K., Wang X., Fang T., Xu P., Zhu L., Li D. (2019). Source and potential risk assessment of suspended atmospheric microplastics in Shanghai. Sci. Total Environ..

[76] Chandrakanthan K., Fraser M.P., Herckes P. (2023). Airborne microplastics in a suburban location in the desert southwest: Occurrence and identification challenges. Atmos. Environ..

[77] Li J., Zhang J., Ren S., Huang D., Liu F., Li Z., Zhang H., Zhao M., Cao Y., Mofolo S. (2023). Atmospheric deposition of microplastics in a rural region of North China Plain. Sci. Total Environ..

[78] Fan Y., Zheng J., Xu W., Zhang Q., Chen N., Wang H., Qian X., Wang G. (2024). Spatiotemporal occurrence and characteristics of microplastics in the urban road dust in a megacity, eastern China. J. Hazard. Mater..

[79] Narmadha V.V., Jose J., Patil S., Farooqui M.O., Srimuruganandam B., Saravanadevi S., Krishnamurthi K. (2020). Assessment of Microplastics in Roadside Suspended Dust from Urban and Rural Environment of Nagpur, India. Int. J. Environ. Res..

[80] Romarate R.A., Ancla S.M.B., Patilan D.M.M., Inocente S.A.T., Pacilan C.J.M., Sinco A.L., Guihawan J.Q., Capangpangan R.Y., Lubguban A.A., Bacosa H.P. (2023). Breathing plastics in Metro Manila, Philippines: Presence of suspended atmospheric microplastics in ambient air. Environ. Sci. Pollut. Res..

[81] Luo D., Wang Z., Liao Z., Chen G., Ji X., Sang Y., Qu L., Chen Z., Wang Z., Dahlgren R.A. (2024). Airborne microplastics in urban, rural and wildland environments on the Tibetan Plateau. J. Hazard. Mater..

[82] Aves A., Ruffell H., Evangeliou N., Gaw S., Revell L.E. (2024). Modelled sources of airborne microplastics collected at a remote Southern Hemisphere site. Atmos. Environ..

[83] Kyriakoudes G., Turner A. (2023). Suspended and deposited microplastics in the coastal atmosphere of southwest England. Chemosphere.

[84] Abbasi S., Turner A. (2021). Dry and wet deposition of microplastics in a semi-arid region (Shiraz, Iran). Sci. Total Environ..

[85] ATSDR (2016). Exposure Dose Guidance for Determining Life Expectancy and Exposure Factor. US Department of Health and Human Services, Public Health Service. https://www.atsdr.cdc.gov/pha-guidance/resources/ATSDR-EDG-Life-Expectancy-Exposure-Factor-508.pdf.

[86] EPA (2011). Exposure Factors Handbook, Chapter 6. Exposure Factors Handbook: 2011 Edition. The Exposure Factors Handbook Provides Information on Various Physiological and Behavioral Factors Commonly Used in Assessing Exposure to Environmental Chemicals. https://www.epa.gov/sites/default/files/2015-09/documents/efh-chapter06.pdf.

[87] EPA (2011). Exposure Factors Handbook, Chapter 8. Exposure Factors Handbook: 2011 Edition. The Exposure Factors Handbook Provides Information on Various Physiological and Behavioral Factors Commonly Used in Assessing Exposure to Environmental Chemicals. https://www.epa.gov/sites/default/files/2015-09/documents/efh-chapter08.pdf.

[88] Jiajia Z., Guoyuan Z., Xuexia W., Wencheng D., Li X., Baoyin L., Yunsen M., Xuran Z., Lianjie S., Chen Y. (2021). Exploring the Occurrence Characteristics of Microplastics in Typical Maize Farmland Soils with Long-Term Plastic Film Mulching in Northern China. Front. Mar. Sci..

[89] Miao H., Zhang S., Gao W., Zhou J., Cai H., Wu L., Liu J., Wang Z., Liu T. (2024). Microplastics occurrence and distribution characteristics in mulched agricultural soils of Guizhou province. Sci. Rep..

[90] Zhang Z., Zhang F., Yang X., Zhang J. (2024). The occurrence and distributions characteristics of microplastics in soils of different land use patterns in Karst Plateau, Southwest China. Sci. Total Environ..

[91] Zhou Y., Wang J., Zou M., Yin Q., Qiu Y., Li C., Ye B., Guo T., Jia Z., Li Y. (2022). Microplastics in urban soils of Nanjing in eastern China: Occurrence, relationships, and sources. Chemosphere.

[92] Zhu B., Chen Y., Jiang L., Liu C., Zhu H., Qiu D., Wang S. (2023). Quantification and characterization of microplastics in farmland soils of Jiangsu Province, East China. Environ. Sci. Pollut. Res..

[93] Haixin Z., Yimei H., Shaoshan A., Haohao L., Xiaoqian D., Pan W., Mengyuan F. (2022). Land-use patterns determine the distribution of soil microplastics in typical agricultural areas on the eastern Qinghai-Tibetan Plateau. J. Hazard. Mater..

[94] Ye C., Lin J., Li Z., Wang G., Li Z. (2024). Characteristics of Microplastic Pollution in Agricultural Soils in Xiangtan, China. Sustainability.

[95] Medyńska-Juraszek A., Szczepańska A. (2023). Microplastic Pollution in EU Farmland Soils: Preliminary Findings from Agricultural Soils (Southwestern Poland). Agriculture.

[96] van den Berg P., Huerta-Lwanga E., Corradini F., Geissen V. (2020). Sewage sludge application as a vehicle for microplastics in eastern Spanish agricultural soils. Environ. Pollut..

[97] Harms I.K., Diekötter T., Troegel S., Lenz M. (2021). Amount distribution and composition of large microplastics in typical agricultural soils in Northern Germany. Sci. Total Environ..

[98] Billings A., Carter H., Cross R.K., Jones K.C., Pereira M.G., Spurgeon D.J. (2023). Co-occurrence of macroplastics, microplastics, and legacy and emerging plasticisers in UK soils. Sci. Total Environ..

[99] Leitão I.A., van Schaik L., Ferreira A.J.D., Alexandre N., Geissen V. (2023). The spatial distribution of microplastics in topsoils of an urban environment–Coimbra city case-study. Environ. Res..

[100] Cheng Y.-L., Zhang R., Tisinger L., Cali S., Yu Z., Chen H.Y., Li A. (2021). Characterization of microplastics in sediment using stereomicroscopy and laser direct infrared (LDIR) spectroscopy. Gondwana Res..

[101] Ren X., Ge J., Wei Z., Zhang W., Wen H. (2023). The Occurrence and Characteristics of Microplastic Pollution in the Agricultural Soils of Anhui Province, in Eastern China. Water Air Soil Pollut..

[102] Nematollahi M.J., Keshavarzi B., Mohit F., Moore F., Busquets R. (2022). Microplastic occurrence in urban and industrial soils of Ahvaz metropolis: A city with a sustained record of air pollution. Sci. Total Environ..

[103] Li W., Wang S., Wufuer R., Duo J., Pan X. (2023). Distinct soil microplastic distributions under various farmland-use types around Urumqi, China. Sci. Total Environ..

[104] Corradini F., Meza P., Eguiluz R., Casado F., Huerta-Lwanga E., Geissen V. (2019). Evidence of microplastic accumulation in agricultural soils from sewage sludge disposal. Sci. Total Environ..

[105] Bi D., Wang B., Li Z., Zhang Y., Ke X., Huang C., Liu W., Luo Y., Christie P., Wu L. (2023). Occurrence and distribution of microplastics in coastal plain soils under three land-use types. Sci. Total Environ..

[106] Nizzetto L., Futter M., Langaas S. (2016). Are Agricultural Soils Dumps for Microplastics of Urban Origin?. Environ. Sci. Technol..

[107] Perfetti-Bolaño A., Araneda A., Muñoz K., Barra R.O. (2022). Occurrence and Distribution of Microplastics in Soils and Intertidal Sediments at Fildes Bay, Maritime Antarctica. Front. Mar. Sci..

[108] Gröndahl F., Sidenmark J., Thomsen A. (2009). Survey of waste water disposal practices at Antarctic research stations. Polar Res..

[109] Huang H., Mohamed B.A., Li L.Y. (2023). Accumulation and fate of microplastics in soils after application of biosolids on land: A review. Environ. Chem. Lett..

[110] Jenner L.C., Rotchell J.M., Bennett R.T., Cowen M., Tentzeris V., Sadofsky L.R. (2022). Detection of microplastics in human lung tissue using μFTIR spectroscopy. Sci. Total Environ..

[111] Huang S., Huang X., Bi R., Guo Q., Yu X., Zeng Q., Huang Z., Liu T., Wu H., Chen Y. (2022). Detection and Analysis of Microplastics in Human Sputum. Environ. Sci. Technol..

[112] Ho Y.-W., Lim J.Y., Yeoh Y.K., Chiou J.-C., Zhu Y., Lai K.P., Li L., Chan P.K.S., Fang J.K.-H. (2022). Preliminary Findings of the High Quantity of Microplastics in Faeces of Hong Kong Residents. Toxics.

[113] Ibrahim Y., Tuan Anuar S., Azmi A., Wan Mohd Khalik W., Lehata S., Hamzah S., Ismail D., Ma Z., Dzulkarnaen A., Zakaria Z. (2021). Detection of microplastics in human colectomy specimens. JGH Open.

[114] Leonard S.V.L., Liddle C.R., Atherall C.A., Chapman E., Watkins M., Calaminus S.D.J., Rotchell J.M. (2024). Microplastics in human blood: Polymer types, concentrations and characterisation using μFTIR. Environ. Int..

[115] Yang Y., Xie E., Du Z., Peng Z., Han Z., Li L., Zhao R., Qin Y., Xue M., Li F. (2023). Detection of Various Microplastics in Patients Undergoing Cardiac Surgery. Environ. Sci. Technol..

[116] Zhong Y., Feng Y., Huang Y., Wang B., Shi W., Liang B., Li Z., Zhang B., Du J., Xiu J. (2024). Polystyrene nanoplastics accelerate atherosclerosis: Unraveling the impact on smooth muscle cells through KIF15-mediated migration. Ecotoxicol. Environ. Saf..

[117] Huo C., Zhu Y., Fang X., Cui J., Ye H., Zhao H., Ye L., Zhou L. (2024). Polystyrene Microplastics Induce Injury to the Vascular Endothelial Through NLRP3-Mediated Pyroptosis. Environ. Toxicol..

[118] Zhang M., Shi J., Pan H., Zhu J., Wang X., Song L., Deng H. (2024). A novel tiRNA-Glu-CTC induces nanoplastics accelerated vascular smooth muscle cell phenotypic switching and vascular injury through mitochondrial damage. Sci. Total Environ..

[119] Zhou Y., Wu Q., Li Y., Feng Y., Wang Y., Cheng W. (2023). Low-dose of polystyrene microplastics induce cardiotoxicity in mice and human-originated cardiac organoids. Environ. Int..

[120] Wang L., Pei W., Li J., Feng Y., Gao X., Jiang P., Wu Q., Li L. (2024). Microplastics induced apoptosis in macrophages by promoting ROS generation and altering metabolic profiles. Ecotoxicol. Environ. Saf..

[121] Liu S., Wang C., Yang Y., Du Z., Li L., Zhang M., Ni S., Yue Z., Yang K., Wang Y. (2024). Microplastics in three types of human arteries detected by pyrolysis-gas chromatography/mass spectrometry (Py-GC/MS). J. Hazard. Mater..

[122] Marfella R., Prattichizzo F., Sardu C., Fulgenzi G., Graciotti L., Spadoni T., D’Onofrio N., Scisciola L., La Grotta R., Frigé C. (2024). Microplastics and Nanoplastics in Atheromas and Cardiovascular Events. N. Engl. J. Med..

[123] Wu D., Feng Y., Wang R., Jiang J., Guan Q., Yang X., Wei H., Xia Y., Luo Y. (2023). Pigment microparticles and microplastics found in human thrombi based on Raman spectral evidence. J. Adv. Res..

[124] Yang Y., Zhang F., Jiang Z., Du Z., Liu S., Zhang M., Jin Y., Qin Y., Yang X., Wang C. (2024). Microplastics are associated with elevated atherosclerotic risk and increased vascular complexity in acute coronary syndrome patients. Part. Fibre Toxicol..

[125] Teng J., Zhao J., Zhu X., Shan E., Zhang C., Zhang W., Wang Q. (2021). Toxic effects of exposure to microplastics with environmentally relevant shapes and concentrations: Accumulation, energy metabolism and tissue damage in oyster *Crassostrea gigas*. Environ. Pollut..

[126] Deng Y., Yang P., Tan H., Shen R., Chen D. (2023). Polylactic Acid Microplastics Do Not Exhibit Lower Biological Toxicity in Growing Mice Compared to Polyvinyl Chloride Microplastics. J. Agric. Food Chem..

[127] Jing L., Zhang Y., Zhang Q., Zhao H. (2024). Polystyrene microplastics disrupted physical barriers, microbiota composition and immune responses in the cecum of developmental Japanese quails. J. Environ. Sci..

[128] Khan A., Jia Z. (2023). Recent insights into uptake, toxicity, and molecular targets of microplastics and nanoplastics relevant to human health impacts. iScience.

[129] Kim J.E., Sonar N.S., Thakuri L.S., Park J.W., Kim K.-T., Rhyu D.Y. (2025). Mixtures of polystyrene micro and nanoplastics affects fat and glucose metabolism in 3T3-L1 adipocytes and zebrafish larvae. NanoImpact.

[130] Tao J., Deng P., Lin M., Chen C., Ma Q., Yang L., Zhang W., Luo Y., Chen S., Pi H. (2024). Long-term exposure to polystyrene microplastics induces hepatotoxicity by altering lipid signatures in C57BL/6J mice. Chemosphere.

[131] Luo T., Wang C., Pan Z., Jin C., Fu Z., Jin Y. (2019). Maternal Polystyrene Microplastic Exposure during Gestation and Lactation Altered Metabolic Homeostasis in the Dams and Their F1 and F2 Offspring. Environ. Sci. Technol..

[132] Wang C., Hou M., Shang K., Wang H., Wang J. (2022). Microplastics (Polystyrene) Exposure Induces Metabolic Changes in the Liver of Rare Minnow (*Gobiocypris rarus*). Molecules.

[133] Zhong G., Rao G., Tang L., Wu S., Tang Z., Huang R., Ruan Z., Hu L. (2022). Combined effect of arsenic and polystyrene-nanoplastics at environmentally relevant concentrations in mice liver: Activation of apoptosis, pyroptosis and excessive autophagy. Chemosphere.

[134] Mu Y., Sun J., Li Z., Zhang W., Liu Z., Li C., Peng C., Cui G., Shao H., Du Z. (2022). Activation of pyroptosis and ferroptosis is involved in the hepatotoxicity induced by polystyrene microplastics in mice. Chemosphere.

[135] Zhang N., Li Y.B., He H.R., Zhang J.F., Ma G.S. (2021). You are what you eat: Microplastics in the feces of young men living in Beijing. Sci. Total Environ..

[136] Zhang J., Wang L., Trasande L., Kannan K. (2021). Occurrence of Polyethylene Terephthalate and Polycarbonate Microplastics in Infant and Adult Feces. Environ. Sci. Technol. Lett..

[137] Zhu L., Kang Y., Ma M., Wu Z., Zhang L., Hu R., Xu Q., Zhu J., Gu X., An L. (2024). Tissue accumulation of microplastics and potential health risks in human. Sci. Total Environ..

[138] Horvatits T., Tamminga M., Liu B., Sebode M., Carambia A., Fischer L., Püschel K., Huber S., Fischer E.K. (2022). Microplastics detected in cirrhotic liver tissue. eBioMedicine.

[139] Chen W., Fryrear D.W. (2001). Aerodynamic and Geometric Diameters of Airborne Particles. J. Sediment. Res..

[140] Kelly F.J., Fussell J.C. (2012). Size, source and chemical composition as determinants of toxicity attributable to ambient particulate matter. Atmos. Environ..

[141] Shi W., Cao Y., Chai X., Zhao Q., Geng Y., Liu D., Tian S. (2022). Potential health risks of the interaction of microplastics and lung surfactant. J. Hazard. Mater..

[142] Kang H., Huang D., Zhang W., Wang J., Liu Z., Wang Z., Jiang G., Gao A. (2024). Inhaled polystyrene microplastics impaired lung function through pulmonary flora/TLR4-mediated iron homeostasis imbalance. Sci. Total Environ..

[143] Zhang J., Du J., Liu D., Zhuo J., Chu L., Li Y., Gao L., Xu M., Chen W., Huang W. (2024). Polystyrene microplastics induce pulmonary fibrosis by promoting alveolar epithelial cell ferroptosis through cGAS/STING signaling. Ecotoxicol. Environ. Saf..

[144] Han Y., Ye L., Du F., Ye M., Li C., Zhu X., Wang Q., Jiang H., Liu Z., Ma J. (2021). Iron metabolism regulation of epithelial-mesenchymal transition in idiopathic pulmonary fibrosis. Ann. Transl. Med..

[145] Jin W., Zhang W., Tang H., Wang P., Zhang Y., Liu S., Qiu J., Chen H., Wang L., Wang R. (2024). Microplastics exposure causes the senescence of human lung epithelial cells and mouse lungs by inducing ROS signaling. Environ. Int..

[146] Liu S., Zheng J., Lan W., Yang Z., Li M., Li J., Yu J., Yang S., Du J., Dong R. (2025). Microplastics exposed by respiratory tract and exacerbation of community-acquired pneumonia: The potential influences of respiratory microbiota and inflammatory factors. Environ. Int..

[147] Tuna A., Taş B.M., Başaran Kankılıç G., Koçak F.M., Şencan Z., Cömert E., Bayar Muluk N., Kaçmaz B., Gül S., Böke E. (2023). Detection of microplastics in patients with allergic rhinitis. Eur. Arch. Oto-Rhino-Laryngol..

[148] Chen Q., Gao J., Yu H., Su H., Yang Y., Cao Y., Zhang Q., Ren Y., Hollert H., Shi H. (2022). An emerging role of microplastics in the etiology of lung ground glass nodules. Environ. Sci. Eur..

[149] Lee Y., Heo S.-E., Park K., Yong H., Lee Y., Kim T., Noh Y., Choi W., Choi B., Kim D. (2025). Amplified lung cancer hazards from surface defects in aged polypropylene microplastics. Chem. Eng. J..

[150] Xia X., Guo W., Ma X., Liang N., Duan X., Zhang P., Zhang Y., Chang Z., Zhang X. (2023). Reproductive toxicity and cross-generational effect of polyethylene microplastics in *Paramisgurnus dabryanus*. Chemosphere.

[151] Wang Z., Zhang R., Zhang Y., Xiong Y., Zhang M. (2025). The risk of short-term microplastic exposure on female reproductive function: A rat model study. NanoImpact.

[152] Garcia M.A., Liu R., Nihart A., El Hayek E., Castillo E., Barrozo E.R., Suter M.A., Bleske B., Scott J., Forsythe K. (2024). Quantitation and identification of microplastics accumulation in human placental specimens using pyrolysis gas chromatography mass spectrometry. Toxicol. Sci..

[153] Park E.-J., Han J.-S., Park E.-J., Seong E., Lee G.-H., Kim D.-W., Son H.-Y., Han H.-Y., Lee B.-S. (2020). Repeated-oral dose toxicity of polyethylene microplastics and the possible implications on reproduction and development of the next generation. Toxicol. Lett..

[154] Tian L., Zhang Y., Chen J., Liu X., Nie H., Li K., Liu H., Lai W., Shi Y., Xi Z. (2024). Effects of nanoplastic exposure during pregnancy and lactation on neurodevelopment of rat offspring. J. Hazard. Mater..

[155] Wang W., Guan J., Feng Y., Nie L., Xu Y., Xu H., Fu F. (2022). Polystyrene microplastics induced nephrotoxicity associated with oxidative stress, inflammation, and endoplasmic reticulum stress in juvenile rats. Front. Nutr..

[156] Wang X., Jian S., Zhang S., Wu D., Wang J., Gao M., Sheng J., Hong Y. (2022). Enrichment of polystyrene microplastics induces histological damage, oxidative stress, Keap1-Nrf2 signaling pathway-related gene expression in loach juveniles (*Paramisgurnus dabryanus*). Ecotoxicol. Environ. Saf..

[157] Xu R., Hua X., Rui Q., Wang D. (2022). Alteration in Wnt signaling mediates induction of transgenerational toxicity of polystyrene nanoplastics in *C. elegans*. NanoImpact.

[158] Shen R., Yang K., Cheng X., Guo C., Xing X., Sun H., Liu D., Liu X., Wang D. (2022). Accumulation of polystyrene microplastics induces liver fibrosis by activating cGAS/STING pathway. Environ. Pollut..

[159] Amereh F., Eslami A., Fazelipour S., Rafiee M., Zibaii M.I., Babaei M. (2019). Thyroid endocrine status and biochemical stress responses in adult male Wistar rats chronically exposed to pristine polystyrene nanoplastics. Toxicol. Res..

[160] Shu Q., Xie S., Junaid M., Zheng R., Tang H., Zou J., Zhou A. (2024). MPs and PFOS single and combined exposure significantly alter genetic expressions of growth hormone and insulin growth factor-related biomarkers during zebrafish embryonic development. Sci. Total Environ..

[161] Ge Q., Zheng T., Ding P., Li Z., Lin X., Li X., He M., Hu G. (2025). Aged microplastics-induced growth inhibition via DNA damage, GH/IGF-1 and HPT axes disruption in zebrafish larvae. Sci. Total Environ..

