# Supplementary material for: Environmental Micro(nano)plastic Exposure and Associated Human Health Risks: A Comprehensive Review
Source: Toxics. 2026 May 18;14(5):442. doi: 10.3390/toxics14050442 (PMC13211621; doi:10.3390/toxics14050442)
Supplement: Supplementary file 1 [file toxics-14-00442-s001.zip › toxics-4258265-supplementary.pdf]

Environmental Microplastic Exposure and Associated Human Health Risks: A Comprehensive Review

Weike Hu[a,1], Dongling Liu[b,1], Zhiying Qiu[a], Jianing Wang[a], Xia Huo[c], Xiang Zeng[a,d,*]

[a] School of Public Health, Zhejiang Chinese Medical University, 548 Binwen Road, Hangzhou 310053, Zhejiang Province, China

[b] School of Basic Medical Science, Zhejiang Chinese Medical University, 548 Binwen Road, Hangzhou 310053, Zhejiang Province, China

[c] Laboratory of Environmental Medicine and Developmental Toxicology, Guangdong Key Laboratory of Environmental Pollution and Health, School of Environment, Jinan University, Guangzhou 511443, Guangdong, China

[d] Zhejiang International Science and Technology Cooperation Base of Air Pollution and Health, 548 Binwen Road, Hangzhou 310053, Zhejiang Province, China.

*Correspondence to Xiang Zeng, School of Public Health, Zhejiang Chinese Medical University, Hangzhou 310053, Zhejiang Province, China (Telephone: +86-(0)571-86603107; Email: x.zeng@zcmu.edu.cn, X. Zeng).

[1] These authors contributed equally to this work.

Supplementary Materials

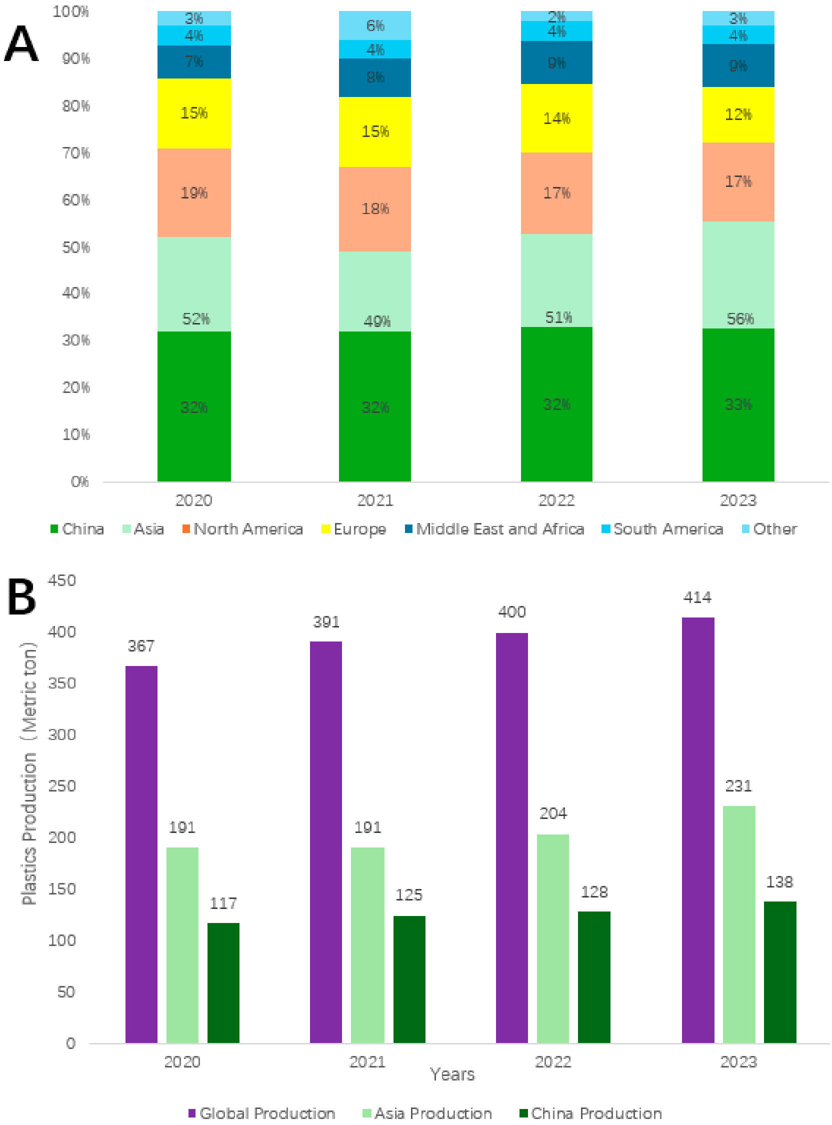

Figure S1. Proportion and Production of plastic products in different regions around the world from 2020 to 2023. (**Figure S1A:** Proportion of total output of plastic products in different regions from 2020 to 2023; **Figure S1B:** Production of plastic products in China, Asia, and the world from 2020 to 2023).

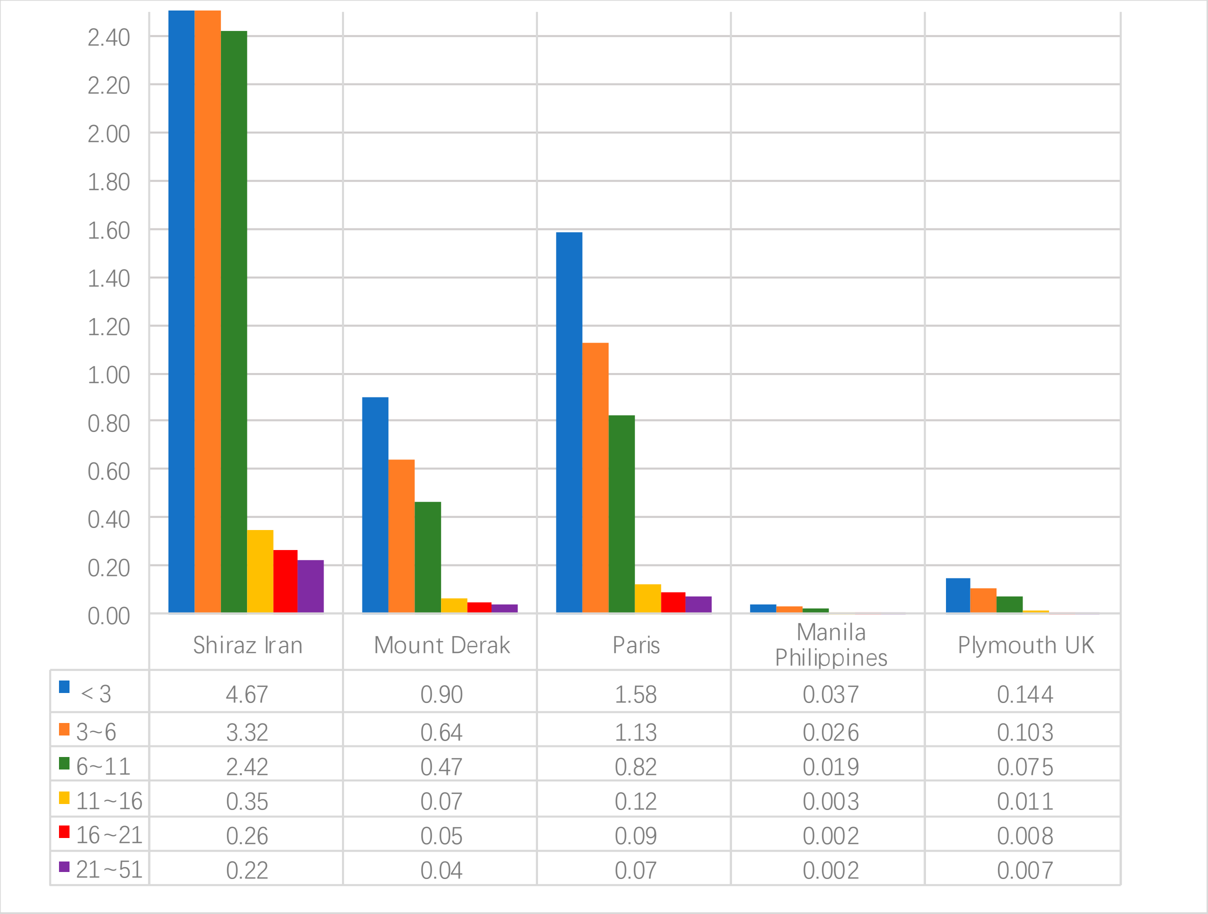

	Shiraz Iran	Mount Derak	Paris	Manila Philippines	Plymouth UK
< 3	4.67	0.90	1.58	0.037	0.144
3~6	3.32	0.64	1.13	0.026	0.103
6~11	2.42	0.47	0.82	0.019	0.075
11~16	0.35	0.07	0.12	0.003	0.011
16~21	0.26	0.05	0.09	0.002	0.008
21~51	0.22	0.04	0.07	0.002	0.007

Figure S2. Average microplastic exposure doses for different age groups outdoors in different regions (MPs/kg-BW/d).

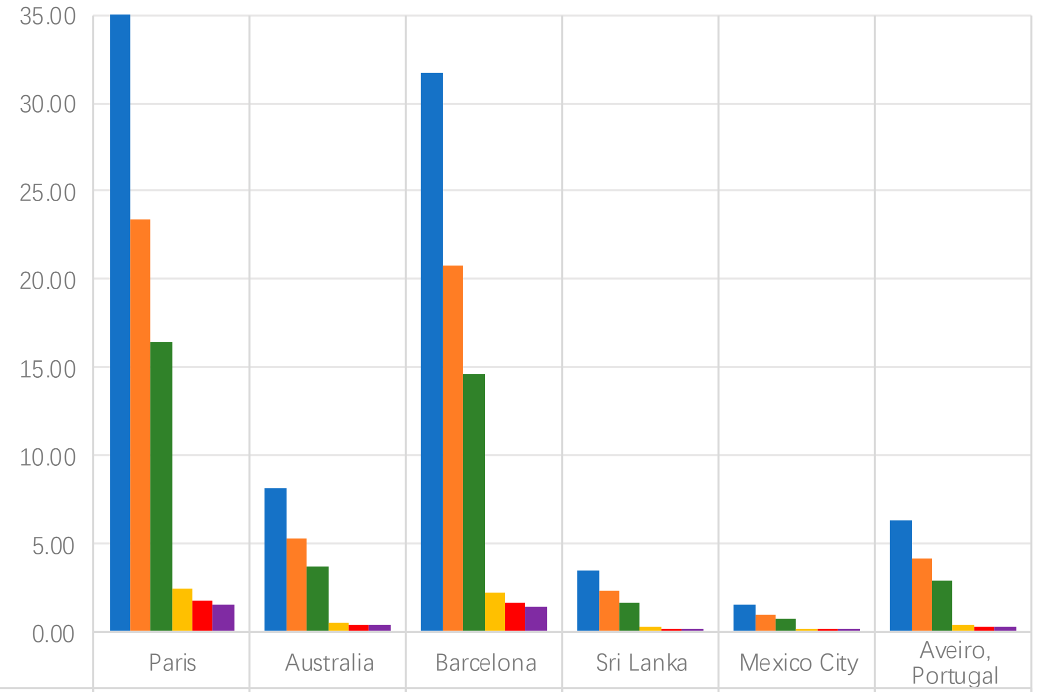

	Paris	Australia	Barcelona	Sri Lanka	Mexico City	Aveiro, Portugal
< 3	35.69	8.10	31.73	3.50	1.51	6.28
3~6	23.35	5.30	20.76	2.29	0.99	4.11
6~11	16.43	3.73	14.61	1.61	0.70	2.89
11~16	2.47	0.56	2.20	0.24	0.10	0.43
16~21	1.81	0.41	1.61	0.18	0.08	0.32
21~51	1.55	0.35	1.38	0.15	0.07	0.27

Figure S3. Average microplastic exposure doses for different age groups indoors in different regions (MPs/kg-BW/d).

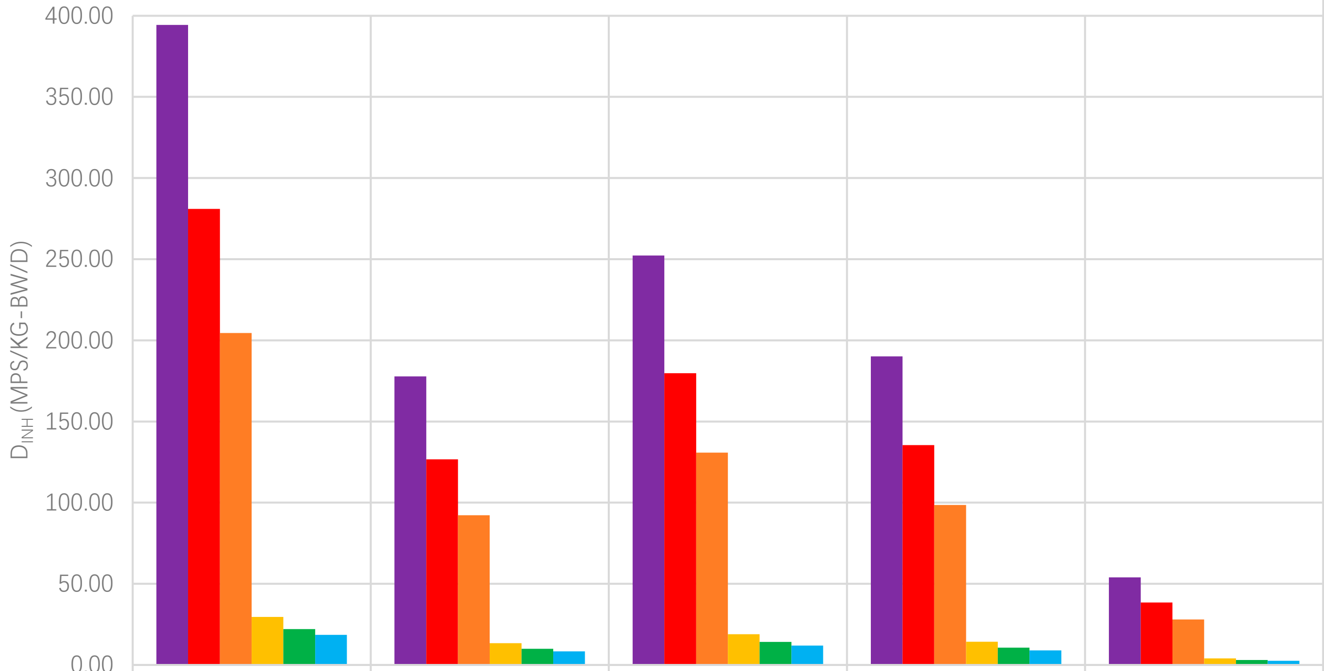

	Wenzhou Urban	Wenzhou Rural	Nanjing	Shanghai	Tibetan plateau
< 3	394.35	177.81	252.28	190.13	53.96
3~6	280.97	126.69	179.74	135.47	38.45
6~11	204.47	92.20	130.81	98.59	27.98
11~16	29.63	13.36	18.96	14.29	4.05
16~21	22.12	9.98	14.15	10.67	3.03
21~51	18.56	8.37	11.88	8.95	2.54

Figure S4. Average microplastic exposure doses for different age groups outdoors of different regions in China (MPs/kg-BW/d).

Table S1. Risk Level Categories of Microplastics.

Method	I	II	III	IV
PLI (Pollution Load Index)	<10	10-100	100-1000	>1000
PHI (Polymer Index)	<10	10-20	20-30	>30

The pollutant load index (PLI) method is a method used to reflect the degree of microplastic pollution in a certain area. Its calculation formula is as follows (1)~(3).

$$C_F = C \div C_0 \quad (1)$$

$$I_{PL,n} = \sqrt{C_F} \quad (2)$$

$$I_{PL,\ Zone} = \sqrt[n]{I_{PL,1} \times I_{PL,2} \times \ldots \times I_{PL,n}} \quad (3)$$

C_F represents the pollution coefficient of the MPs, C represents the measured value of the MPs at a site, C_0 represents the reference value of the MP concentration, $I_{PL,n}$ represents the pollution loading index of the MPs at site n, and $I_{PL,zone}$ represents the pollution loading index of the MPs in the zone.

The polymer hazard index (PHI) method is used to evaluate the ecotoxicological risk of different types of microplastics by using the hazard index of different types of plastic polymers as an evaluation factor. The formula is as follows (1)-(4):

$$C_f = \frac{C}{C_r} \quad (1)$$

$$T_r = \frac{N_i}{C} \times S_i \quad (2)$$

$$E_r = T_r \times C_f \quad (3)$$

$$I_R = \sum_{r=1}^{n} E_r \quad (4)$$

where C_f is the contamination coefficient of MPs at a site, C is the measured concentration of MPs at a site, C_r is the standardized reference value of the concentration of MPs at the rth site, T_r is the ecotoxicity response factor of MPs at the rth site, N_i is the amount of i polymer at a site, S_i is the hazard factor of i polymer, E_r is the potential ecological risk index of MPs at the rth site, and I_R is the regional hazard index of polymer i at a site.

Table S2. Status of soil microplastics in China.

Region	Land type	Sampling depth	Average concentration	Polymer type	Detection Methods	References
Pingquan, Hebei Province, China	Film-covered corn-growing areas	0-10 cm	871 ± 641 Items/kg	PE, PP, PET, PEA,	Stereoscopic	[77]
	Non-film-covered corn-growing areas	0-10 cm	485 ± 257 Items/kg	PAN, Rayon,	Microscope	
Guizhou Province, China	Vegetable Growing Area	30 cm	549.23-2706.37 Items/kg	PE	Metallographic	[78]
Karst Plateau of Guizhou	Industrial mining warehouses	/	3114 Items/kg	PE, PP, PVC, PET	FTIR	[79]
Nanjing, Suzhou, Xuzhou	Urban surface soils	/	461±222 Items/kg	PET, PE	FTIR	[80]
Jiangsu Province, China	Agricultural soil	0-5 cm	3255 ± 1998 Items/kg	PVC	LDIR	[81]
Qinghai province, China	Industrial Land	/	2449.0 Items/kg	/	FTIR	[82]
Xiangtan, Hunan Province,	Rice soil	0-10 cm	1279.89 Items/kg	PE, PP, PS, PA, PVC	FTIR	[83]

Table S3. Status of soil microplastics in Europe.

Region	Land type	Soil depth	Average concentration	Polymer Type	Methods of analysis	References
**Lower Silesia, Poland**	A: Loamy soil farmland	0-25 cm	1540 ± 912 particles/kg	/	Optical Microscope	[84]
	B: sandy soil farmland		383 ± 188 particles/kg			
	C: Powdery clay soil farmland		933 ± 682 particles/kg			
	D: Former floodplain pastureland		1200 ± 234 particles/kg			
	E: Sewage sludge disposal pastures		4050 ± 2831 particles/kg			
	F: Former industrial area, now orchard soils		2116 ± 614 particles/kg			
**Valencia, Spain**	Sewage sludge application to historical	0-10 cm	1730 ± 920 particles/kg	PP, PVC	μFTIR	[85]
	agricultural soils	10-30 cm	1610 ± 920 particles/kg			
	No sewage sludge application to historical	0-10 cm	960 ± 420 particles/kg			
	agricultural soils	10-30 cm	920 ± 480 particles/kg			
**Schleswig-Holstein,**	agricultural soil	0-10 cm	5.8 ± 8 particles/kg	PE, PP, PA, Nylon	FTIR, Stereoscopic	[86]
**Germany**		10-20 cm	3.3 ± 3.1 particles/kg		Microscope	
		20-30 cm	1.9 ± 1.6 particles/kg			
**Midlands, South of England**	Tree-covered woodland	15 cm	2.6 particles/g	PE, PA	FTIR	[87]

	Landfill downwind		12.3 ± 27.5 particles/g			
	City parks		15.7 ± 19.5 particles/g			
	City roadside		17.3 ± 24.1 particles/g			
**City of Coimbra, Portugal**	Forest	0-10 cm	$55\times10^3 \pm 39\times10^3$ particles/kg	PP, PVC, PE, PLA	μFTIR,	[88]
	Green Park		$158\times10^3 \pm 104\times10^3$ particles/kg		Optical Microscope	
	Swamp		$99\times10^3 \pm 21\times10^3$ particles/kg			
	Farms		$104\times10^3 \pm 111\times10^3$ particles/kg			
	Parking lots		$102\times10^3 \pm 42\times10^3$ particles/kg			
	Urban construction area		$92\times10^3 \pm 47\times10^3$ particles/kg			
	Urban Industrial Zone		$127\times10^3 \pm 61\times10^3$ particles/kg			
	Municipal landfills		$150\times10^3 \pm 16\times10^3$ particles/kg			

Abbreviation, PE: Polyethylene; PET: Polyethylene terephthalate; PS: Polystyrene; PP: Polypropylene; PA: Polyamide; PVC: polyvinyl chloride; PEA: Polyetherimides; PLA: Polylactic Acid, PAN: Polyacrylonitrile.
